# Resistive Random Access Memory (RRAM): an Overview of Materials, Switching Mechanism, Performance, Multilevel Cell (mlc) Storage, Modeling, and Applications

**DOI:** 10.1186/s11671-020-03299-9

**Published:** 2020-04-22

**Authors:** Furqan Zahoor, Tun Zainal Azni Zulkifli, Farooq Ahmad Khanday

**Affiliations:** 1 Department of Electrical and Electronics Engineering, Universiti Teknologi Petronas, Seri Iskandar, Perak, 32610 Malaysia; 2 Department of Electrical and Electronics Engineering, Universiti Teknologi Petronas, Seri Iskandar, Perak, 32610 Malaysia; 3 P.G. Department of Electronics and Instrumentation Technology, University of Kashmir, Srinagar, Jammu and Kashmir, 190005 India

**Keywords:** Emerging memory, Multilevel cell (MLC), Non-volatile storage, Oxygen vacancies, Resistive random access memory (RRAM), Resistance switching

## Abstract

In this manuscript, recent progress in the area of resistive random access memory (RRAM) technology which is considered one of the most standout emerging memory technologies owing to its high speed, low cost, enhanced storage density, potential applications in various fields, and excellent scalability is comprehensively reviewed. First, a brief overview of the field of emerging memory technologies is provided. The material properties, resistance switching mechanism, and electrical characteristics of RRAM are discussed. Also, various issues such as endurance, retention, uniformity, and the effect of operating temperature and random telegraph noise (RTN) are elaborated. A discussion on multilevel cell (MLC) storage capability of RRAM, which is attractive for achieving increased storage density and low cost is presented. Different operation schemes to achieve reliable MLC operation along with their physical mechanisms have been provided. In addition, an elaborate description of switching methodologies and current voltage relationships for various popular RRAM models is covered in this work. The prospective applications of RRAM to various fields such as security, neuromorphic computing, and non-volatile logic systems are addressed briefly. The present review article concludes with the discussion on the challenges and future prospects of the RRAM.

## Introduction

Random access memory referred to as RAM can either be volatile or non-volatile. A volatile memory loses its previous stored data on removing the power supply as is the case for dynamic random-access memory (DRAM) and static random-access memory (SRAM). For non-volatile memory, the contents that were stored previously will continue to be retained even after the removal of the supply. Flash memory is a typical example of non-volatile memory. Memory technologies combine the advantages and disadvantages to achieve higher performance, e.g. DRAMs employed in a computer system has high capacity and density, but they are volatile, meaning there is a need to refresh every few milliseconds. Due to this refreshing, the energy consumption of the device increases which is not desirable. SRAM, on the other hand, is fast but it is also volatile just like the DRAM; in addition, SRAM cells are of larger size which hinders its implementation on a large scale. Flash memory, which essentially consists of a metal-oxide-semiconductor field-effect-transistor (MOSFET) in addition to a floating gate in each memory cell, is currently being used extensively particularly for the embedded applications owing to its low cost and high density. Depending upon how memory cells are organized, Flash memory is classified as NOR Flash and NAND Flash [1]. In NOR Flash, cells are read and programmed individually as they are connected in parallel to bit lines. This resembles the parallel connection of transistors in a CMOS NOR gate architecture. For the case of NAND Flash, the architecture resembles that of a CMOS NAND gate as the cells are connected in series to the bit lines. It must be noted that less space is consumed by the series connection as compared to the parallel one which results in a reduced cost of NAND Flash. However, both types of Flash memories suffer from several disadvantages such as low operation speed (write/erase time: 1 ms/0.1 ms), limited endurance (10^6^ write/erase cycles), and high write voltage (> 10 V) [2].

The memory technologies mentioned above, i.e. DRAM, SRAM, and Flash, are charge storage-based memories. DRAM stores the information in the form of charge at the capacitor, and SRAM is based on the storage of charge at the nodes of the cross-coupled inverters, whereas the Flash memory technology uses the floating gate of the transistor to store the charge. All these existing charge storage-based memory technologies are currently facing challenges to scale down to 10 nm node or beyond. This is attributed to the loss of stored charge at nanoscale, which results in the degradation of the performance, reliability, and noise margin. In addition, requirements of large refresh dynamic power for DRAM and leakage power for both SRAM and DRAM pose serious challenges for the design of future memory hierarchy.

Therefore, a new class of memories usually referred to as emerging memory technologies are currently undergoing development and are being actively researched primarily in the industry with the aim to revolutionize the existing memory hierarchy [3]. These emerging memory technologies aim to integrate the switching speed of SRAM, storage density comparable to that of DRAM, and the non-volatility of Flash memory, thus become very attractive alternatives for future memory hierarchy.

To classify a memory device as an ideal one, it should have the following characteristics: low operating voltage (<1 V), long cycling endurance (>10^17^ cycles), enhanced data retention time (>10 years), low energy consumption (fJ/bit), and superior scalability (<10 nm) [4]. However, no single memory to date that satisfies these ideal characteristics. Various emerging memory technologies are actively being investigated to meet a part of these ideal memory characteristics. These memory technologies that depend upon the change of resistance rather than charge to store the information are as follows: (i) phase change memory (PCM), (ii) spin-transfer torque magnetoresistive random access memory (STT-MRAM), and (iii) resistive random access memory (RRAM). In phase change memory, the switching medium consists of a chalcogenide material (commonly Ge_2_-Sb_2_-Te_5_, GST) [5–7]. PCM relies on the difference in resistance between the crystalline phase and amorphous phase for efficient data storage capability. The crystalline phase denotes the low resistance state (LRS) or ON state of the device whereas the amorphous phase denotes the high resistance state (HRS) or OFF state. The SET operation corresponds to LRS generally referred to storing logic value ‘1’, whereas the RESET operation correspond to HRS storing logic value ‘0’ in the device. For SET operation, PCM is heated above its crystallization temperature on the application of voltage pulse, while for RESET operation, a larger electrical current is passed through the cell and then abruptly cut-off so as to melt and then quench the material in order to achieve the amorphous state.

In spin-transfer torque magnetoresistive random access memory, the storage capability is due to the magnetic tunneling junction (MJT) [8–10], which consists of two ferromagnetic layers and a tunneling dielectric sandwiched between them. The magnetic direction of the reference layer is fixed, while the application of external electromagnetic field can change the magnetic direction of the free ferromagnetic layer. If the reference layer and the free layer have the same direction of magnetization, the MTJ is referred to be in the LRS. For MTJ, to be in the HRS, the direction of the magnetization of two ferromagnetic layers is anti-parallel. RRAM consists of an insulating layer (I) sandwiched between the two metal (M) electrodes [11, 12]. RRAM relies on the formation and the rupture of conductive filaments corresponding to LRS and HRS, respectively, in the insulator between two electrodes [13–15].

**Fig. 1:**
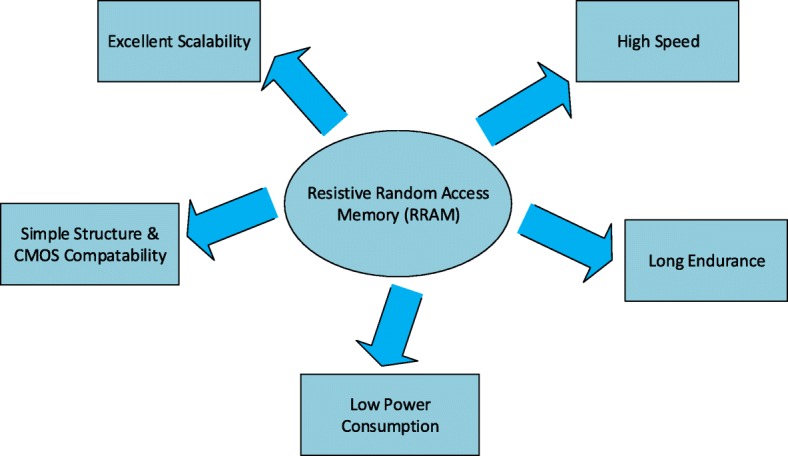
Advantages of RRAM

A detailed comparison of existing and emerging memory technologies is shown in Table 1. As is evident from the table, STT-MRAM and PCM have advantages of a smaller area compared to that of SRAM. While STT-MRAM offers fast write/read speed, long endurance, and low programming voltage, on the other hand, PCM has a disadvantage of extensive write latency. RRAM has a lower programming voltage and faster write/read speed compared to Flash and is seen as potential replacement of Flash memory. Among all the emerging memory technology candidates, RRAM has significant advantages such as easy fabrication, simple metal-insulator-metal (MIM) structure, excellent scalability, nanosecond speed, long data retention, and compatibility with the current CMOS technology, thus offering a competitive solution to future digital memory [16]. The most significant advantages of RRAM are depicted in Fig. 1.

**Table 1:** Comparison of emerging memory technologies

Memory technology	SRAM	DRAM	NAND Flash	NOR Flash	PCM	STT-MRAM	RRAM
Cell area	> 100*F*^2^	6*F*^2^	<4*F*^2^(3D)	10*F*^2^	4– 20*F*^2^	6– 20*F*^2^	<4*F*^2^(3D)
Cell element	6T	1T1C	1T	1T	1T(D)1R	1(2)T1R	1T(D)1R
Voltage	<1 V	<1 V	<10 V	<10 V	<3 V	<2 V	< 3 V
Read time	∼1 ns	∼10 ns	∼10 *μ*s	∼50 ns	<10 ns	<10 ns	< 10 ns
Write time	∼1 ns	∼10 ns	100 *μ*s–1 ms	10 *μ*s–1 ms	∼50 ns	<5 ns	< 10 ns
Write energy (J/bit)	∼fJ	∼10 fJ	∼10 fJ	100 pJ	∼10 pJ	∼0.1 pJ	∼0.1 pJ
Retention	N/A	∼64 ms	>10 y	>10 y	>10 y	>10 y	> 10 y
Endurance	> 10^16^	>10^16^	>10^4^	>10^5^	>10^9^	>10^15^	∼10^6^– 10^12^
Multibit capacity	No	No	Yes	Yes	Yes	Yes	Yes
Non-volatility	No	No	Yes	Yes	Yes	Yes	Yes
Scalability	Yes	Yes	Yes	Yes	Yes	Yes	Yes
F: Feature size of lithography							

**Fig. 2:**
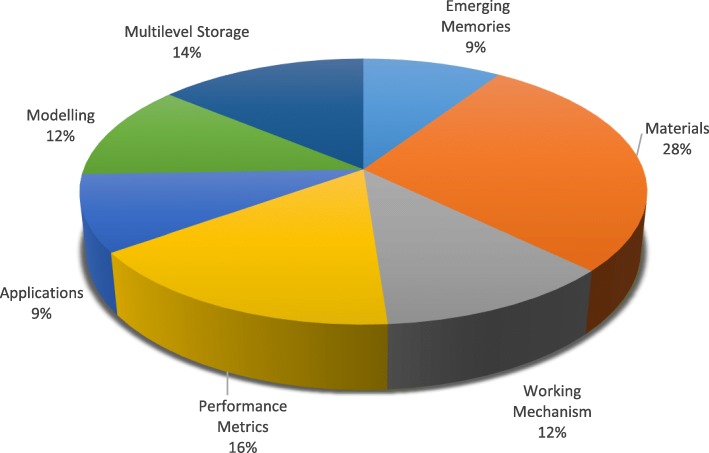
Category wise distribution of papers consulted for preparation of review on RRAM

In this work, recent progress and a detailed overview of RRAM technology are presented. A review of switching materials together with the classification of switching modes and details of the switching mechanism is discussed in the “Resistive Random Access Memory (RRAM)” section. The “Performance Metrics of Resistive Random Access Memory (RRAM)” section highlights various performance metrics of RRAM. Multilevel cell (MLC) characteristics of RRAM along with various MLC operation schemes and their physical mechanisms are analyzed in the “Multilevel Resistive Random Access Memory (RRAM)” section. A detailed discussion on modeling of RRAM device is presented in “Modeling of RRAM Devices” section. In “Applications of RRAM” section various applications of RRAM are discussed. Finally, challenges and future outlook of RRAM is presented in “Challenges and Future Outlook” section. The category wise distribution of papers consulted in the preparation of this review manuscript are presented in Fig. 2.

## Resistive Random Access Memory (RRAM)

### Resistance Switching Materials

It has been observed that in some insulators, the change of resistance occurs under the application of the applied electric field. This property of change of resistance has recently been investigated for developing future non-volatile memories [17]. The resistance switching phenomenon has been observed in a variety of oxides, but binary metal oxides have been extensively studied as a preferred switching material for future non-volatile memory applications primarily due to their compatibility with the CMOS BEOL processing. Various metal-oxide-based materials exhibiting the non-volatile resistance switching such as hafnium oxide (HfO _*x*_) [18–23], titanium oxide (TiO _*x*_) [24–31], tantalum oxide (TaO _*x*_) [32–36], nickel oxide (NiO) [37–40], zinc oxide (ZnO) [41–46], zinc titanate (Zn_2_TiO_4_) [47], manganese oxide (MnO _*x*_) [48, 49], magnesium oxide (MgO) [50], aluminum oxide (AlO _*x*_) [51–53], and zirconium dioxide (ZrO_2_) [54–58] have drawn the most attention and have been studied extensively during the past several years. These metal oxides are deposited usually by pulse laser deposition (PLD), atomic layer deposition (ALD), and reactive sputtering. However, ALD is a widely preferred method owing to its ability to precisely control the thickness and uniformity of the thin film [59].

In conventional electronic devices, the choice of electrode material is important as they act as transport paths for the carriers. For RRAM, the choice of electrode material greatly affects the switching behavior of the device. For example, in copper/poly(3-hexylthiophene): [6,6]-phenyl-C61-butyric acid methyl ester/indium-tin oxide (Cu/P3HT: PCBM/ITO) structure, a stable resistive switching behavior was observed; however, it disappeared after the Cu electrode was replaced with Pt electrode [60]. A wide variety of materials have been utilized as electrodes for RRAM. The electrode materials can be grouped into five categories on the basis of their composition, including elementary substance electrodes, silicon-based electrodes, alloy electrodes, oxide electrodes, and nitrite-based electrodes. The most abundant and the commonly used electrodes are elementary substance electrodes which include Al [51], Ti[49], Cu[30], graphene [61], carbon nanotubes [62], Ag [41], W [36], and Pt [44]. For silicon-based electrodes, p-type Si and n-type Si [63] are the only types of electrodes used. Alloy electrodes usually stabilize the resistive switching behavior and mainly include Cu-Ti [64], Cu-Te[65], and Pt-Al [66]. The most common nitride-based electrodes are the TiN and TaN [67, 68]. The oxide-based electrodes are relatively abundant, including Al-doped ZnO [69], Ga-doped ZnO [70], and ITO [71].

The list of metal oxide materials that have been used recently in the fabrication of the RRAM device along with various combinations of materials used for the top electrode and bottom electrode are shown in Table 2. A detailed comparison of the various parameters is also presented. From the wide variety of materials used, one can predict that the non-volatile resistance switching is widely observed in various types of oxides. The material choice for the fabrication of RRAM gives it an edge as metal oxide metal (MOM) structures can be easily fabricated by making use of oxides currently used in the semiconductor technology. The bottom electrode material in RRAM usually is platinum, which is a bit hard to etch. For single device structure, RRAM can share the same bottom electrode whereas, for the crossbar architecture, the separate bottom electrodes are used for each device. They can be obtained by physical vapor deposition and lift-off successively. The top electrode and the resistive switching layer are deposited either using atomic layer deposition (ALD) or physical vapor deposition (PVD).

**Table 2:** Comparison of various RRAM types

Ref	Year	Top electrode	Oxide material	Bottom electrode	Operation mode	HRS /LRS ratio	Retention	Endurance	*V*_*f*_	*V*_set_	*V*_reset_	*I*_*cc*_
[55]	2007	Ti	ZrO_2_	Pt	Bipolar	NS	NS	>10^4^ cycles	8.8 V	1 V	– 1.5 V	5mA
[46]	2008	Pt	ZnO	Pt	Unipolar	<10^5^	NS	>10^2^ cycles	3.3 V	– 2 V	– 1 V	NS
[81]	2008	TiN	TiO _*x*_/HfO _*x*_	TiN	Bipolar	>10^3^	∼10^5^s	>10^6^ cycles	FF	1.5 V	– 1.4 V	25 *μ*A
[52]	2008	Pt/Ti	Al_2_O_3_	Pt	Bipolar	NS	NS	NS	NS	∼ 7 V	– 2 V	5mA
[39]	2008	Pt	NiO	Pt/Ti	Unipolar	NS	NS	NS	5 V	∼ 1 V	∼3.5 V	1 mA
[30]	2009	Cu	TiO_2_	Pt	Bipolar	∼30	NS	NS	NS	0.8 V	– 1.5 V	300 *μ*A
[58]	2009	Ti	ZrO_2_	Pt	Bipolar	NS	NS	>10^2^ cycles	NS	1 V	– 1.5 V	NS
					Unipolar							
[56]	2009	TiN	ZrO_2_	Pt	Bipolar	NS	NS	>10^2^ cycles	NS	1 V	– 1.5 V	NS
[49]	2009	Ti	MnO_2_	Pt	Bipolar	∼10^2^	>10^4^s	>10^5^ cycles	NS	∼0.7 V	∼– 1.1 V	5mA
[51]	2010	Al/Ti	Al_2_O_3_	Pt	Bipolar	>10	10^4^s	>10^3^ cycles	FF	1.5 V	– 2 V	∼1mA
					Unipolar							
[42]	2010	Pt	ZnO	Pt	Unipolar	∼10^2^	NS	NS	∼ 3.5 V	1.1-2.3 V	0.4-1 V	5mA
[57]	2010	Au	ZrO_2_	Ag	Bipolar	10^4^	∼10^4^s	> 500 cycles	FF	– 0.5 V	0.6 V	1mA
[43]	2011	Au	ZnO	ITO	Bipolar	10^4^	>10^4^s	10^2^ cycles	NS	∼-1.5 V	∼ 0.5V	NS
[82]	2011	TaN	Al_2_O_3_/	Pt	Bipolar	>10^5^	10^5^s	NS	NS	1 V	– 1 V	10mA
			Ru NCs									
[83]	2011	TiN	HfO _*x*_/AlO _*x*_	Pt	Bipolar	NS	NS	10^6^ cycles	∼ 8 V	2.5 V	– 3 V	300 *μ*A
[44]	2012	Pt	ZnO	Pt	Bipolar	10^6^	>10^6^s	>10^6^ cycles	4 V	1.2 V	– 0.5V	3mA
[84]	2013	Ta	TaO _*x*_/TiO_2_	Ti	Bipolar	10^5^	>10^4^s	>10^12^ cycles	FF	5 V	– 4 to -6V	NS
[23]	2014	TiN	HfO_2_	Pt	Bipolar	10^6^	10^4^s	NS	FF	– 4.3 V	6 V	NS
[22]	2015	W/Zr	HfO_2_	TiN	Bipolar	NS	NS	> 10^6^ write cycles	2 V	0.5 V	– 1.25 V	50 *μ*A
								> 10^9^ read cycles				
[32]	2015	Pt	TaO _*x*_	TiN	Bipolar	NS	NS	NS	– 2 V	< 1 V	<– 1 V	50-200 *μ*A
[36]	2015	W	Ta/TaO _*x*_	Pt	Bipolar	> 10^2^	> 10^4^s	> 10^8^ cycles	FF	∼ 0.5 V	∼– 1 V	30-300 *μ*A
[85]	2015	Ti	HfO_2_	TiN	Bipolar	10^5^	10^4^s	10^10^ cycles	NS	3 V	– 3.5 V	1mA
[86]	2015	Ti	Ta_2_O_5_/	Au	Bipolar	2370	NS	< 40 cycles	FF	0.7 V	– 0.7 V	NS
			TiO_2_ NPs									
[24]	2016	ITO	TiO _*x*_/	FTO	Bipolar	10^3^	NS	> 300 cycles	FF	<– 1 V	<0.5 V	20 *μ*A
			Ag NPs									
[26]	2016	Ti/W	TiO _*x*_/MgO	Ru	Bipolar	< 32	NS	>10^3^ write cycles	FF	1.4 V	– 1.8 V	8mA
								>10^9^ read cycles				
[28]	2016	TiN/Ti	TiO _2−*x*_	Au	Bipolar	∼10^5^	NS	10^5^ cycles	FF	1 V	– 1 V	2-200 *μ*A
[41]	2016	Ag	a-ZnO	Pt	Bipolar	>10^7^	>10^6^s	>10^2^ cycles	FF	0.24 V	– 2 V	0.1-0.5 mA
[87]	2016	W	WO_3_/Al_2_O_3_	TiW/Cu	Bipolar	10^4^	NS	∼ 300 cycles	3 V	∼ 3.5 V	∼– 2.5 V	10 *μ*A
[27]	2017	TiN/Ti	TiO _2−*x*_	Au	Bipolar	<10^3^	>10^5^s	> 50 cycles	FF	<– 0.5 V	<1 V	1-200nA
[88]	2017	Al	HfO _*x*_	Al	Unipolar	∼10^4^	NS	NS	∼1V	1.8 V	0.8 V	1 *μ*A-1mA
[89]	2017	Au/Ti	TiO _2−*x*_	Au	Bipolar	>10^4^	NS	NS	FF	1 V	– 1 V	5 *μ*A-1mA
[18]	2018	Ti	HfO_2_	TiN	Bipolar	>10	10^4^s	> 10 ^7^ cycles	FF	0.5 V	– 0.5 V	NS
[31]	2018	ITO	a-TiO_2_	Pt	Bipolar	>10	<10^3^s	NS	4.15 V	0.6 V	– 0.5 V	< 200 *μ*A
[47]	2018	ITO	Zn_2_TiO_4_	Pt	Bipolar	NS	> 10^4^s	> 500 cycles	25 V	0.6 V	– 0.6 V	1-10 mA
[72]	2018	Ag	MnO/Ta_2_O_5_	Pt	Bipolar	10^6^	∼10^4^s	100 cycles	FF	0.8 V	– 1.1 V	1 mA
[90]	2018	Pd	HfO _*x*_/	TiN	Bipolar	10^3^	>10^4^s	>10^8^ cycles	5.25 V	2.2 V	– 2.2 V	100 *μ*A
			Ag NPs									

### Resistance Switching Modes

**Fig. 3:**
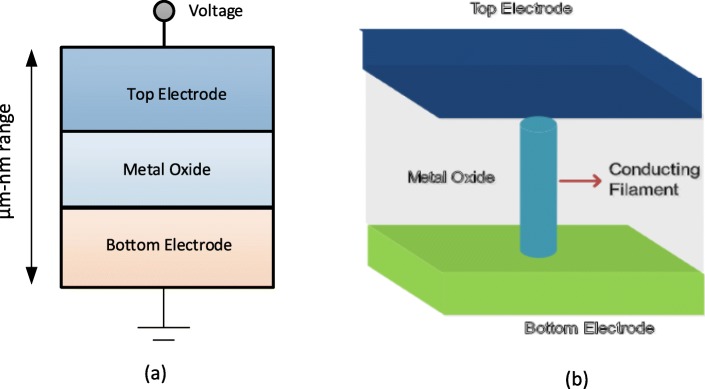
**a** Schematic of metal-insulator-metal structure for RRAM. **b** Cross-sectional view of RRAM

A resistive random access memory (RRAM) consists of a resistive switching memory cell having a metal-insulator-metal structure generally referred to as MIM structure. The structure comprises of an insulating layer (I) sandwiched between the two metal (M) electrodes. The schematic and the cross-sectional view of a RRAM cell is shown in Fig. 3a and b, respectively.

**Fig. 4:**
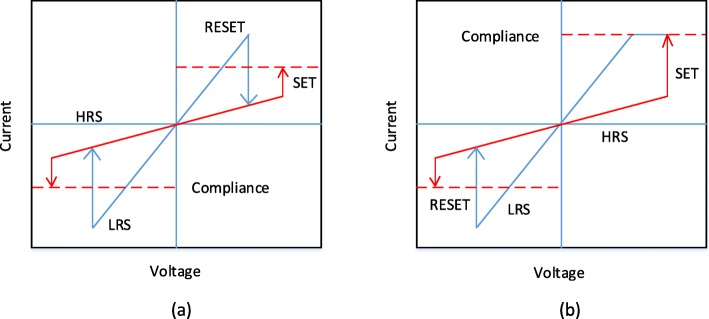
I-V curves for RRAM. **a** Unipolar switching and **b** bipolar switching [4]

The application of the external voltage pulse across the RRAM cell enables a transition of the device from a high resistance state (HRS), or OFF state generally referred as logic value ‘0’ to a low resistance state (LRS), or ON state generally referred as logic value ‘1’ and vice versa. The resistive switching (RS) phenomenon is considered to be the reason behind this change of resistance values in a RRAM cell. An as-prepared RRAM is initially in the high resistance state (HRS), to switch the device from the HRS to the LRS, the application of the high voltage pulse enables the formation of conductive paths in the switching layer and the RRAM cell is switched into a LRS [72]. This process which occurs due to the soft breakdown of the metal insulator metal (MIM) structure is usually referred to as ‘electroforming’ and the voltage at which this process occurs is referred to as forming voltage (*V*_*f*_). It must be noted that the forming voltage is found to be dependent on the cell area [73] and oxide thickness [74]. Now, to switch the RRAM cell from the LRS to HRS, the voltage pulse referred to as the RESET voltage (*V*_reset_) is applied which enables this switching transition and the process is referred to the ‘RESET’ process [75–78]. The HRS of the RRAM can be changed to LRS on the application of the voltage pulse. The voltage at which the transition occurs from HRS to LRS is referred to as SET voltage (*V*_set_) and the process is referred to as the ‘SET’ process [79]. To efficiently read data from RRAM cell, a small read voltage which will not disturb the current state of the cell is applied to determine whether the cell is in logic 0 (HRS) or the logic 1 (LRS) state. Since both LRS and HRS retain their respective values even after the removal of applied voltage, RRAM is a non-volatile memory. Depending on the polarity of the applied voltage, the RRAM can be classified into two types of switching modes: (i) unipolar switching and (ii) bipolar switching [80]. In unipolar switching, the switching (set and reset process) of the device between various resistance states does not depend on the polarity of the applied voltage, i.e. switching can occur on applying a voltage of the same polarity but different magnitude as shown in Fig. 4a. In bipolar switching, on the other hand, the switching (set and reset process) of the device between various resistance states depends on the polarity of the applied voltage, i.e. a transition from a HRS to LRS, occurs at one polarity (either positive or negative) and the opposite polarity switches the RRAM cell back into the HRS as depicted in Fig. 4b. In unipolar switching, Joule heating is interpreted as the physical mechanism responsible to rupture a conducting filament during reset operation. In bipolar switching, on the other hand, the migration of charged species is the main driving force for conductive filament dissolution although Joule heating still contributes to accelerate the migration. In order to ensure, there is no permanent breakdown of the dielectric switching layer during the forming/set process of RRAM, a compliance current (*I*_cc_) is enforced for the RRAM device. The compliance current (*I*_cc_) is ensured usually by a cell selection device (transistor, diode, resistor) or by a semiconductor parameter analyzer during the off-chip testing.

### Resistive Switching Mechanism

The switching of the RRAM cell is based on the growth of conductive filament (CF) inside a dielectric. The CF is a channel having a very less diameter of the order of nanometers which connects the top and the bottom electrodes of the memory cell. A low resistance state (LRS) with high conductivity is obtained when the filament is connected and the high resistance (HRS) results when the filament is disconnected with a gap between the electrodes [91]. Based on the composition of the conductive filament, RRAM can be classified into the following two types: (i) metal ion-based RRAM also referred to as conductive bridge random access memory (CBRAM) and (ii) oxygen vacancies filament-based RRAM referred to as the ‘OxRRAM’. It must be noted here that CBRAM is sometimes referred to as the electrochemical metallization memory (ECM), whereas ‘OxRRAM’ is sometimes also known as valence change memory (VCM).

In metal ion-based RRAM also referred to as ‘CBRAM’, the physical mechanism that is responsible for resistive switching is based on the migration of metal ions and subsequent reduction/oxidation (redox) reactions [92, 93]. The CBRAM structure consists of an oxidizable top electrode (anode) such as Ag, Cu, and Ni, a relatively inert bottom electrode (cathode), e.g. W, Pt, and a sandwiched metal oxide layer between the two electrodes. The filament formation in such memory cells occurs due to the dissolution of the active metal electrodes (most commonly Ag or Cu), the transport of cations (Cu ^+^ or Ag ^+^), and their subsequent deposition or reduction at the inert bottom electrode [94]. Thus, the resistive switching behavior of this type of RRAM is dominated by the formation and dissolution of the metal filaments.

**Fig. 5:**
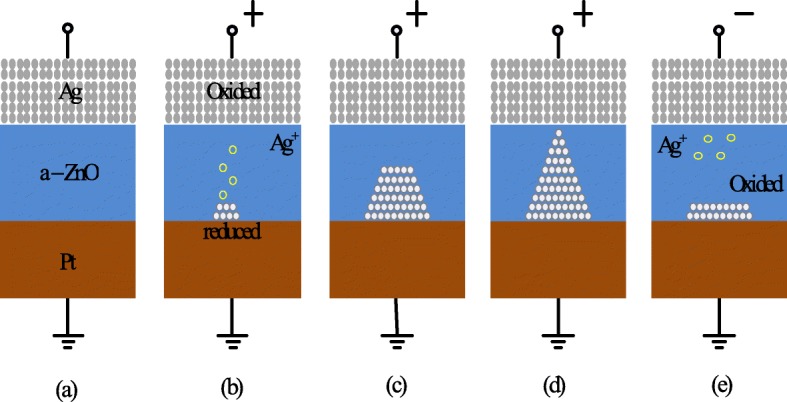
Schematic of the switching mechanism of conductive bridge RRAM. **a** Pristine state of the RRAM device. **b**, **c** Oxidation of Ag and migration of Ag ^+^ cations towards cathode and their reduction. **d** Accumulation of Ag atoms and Pt electrode leads to growth of highly conductive filament. **e** Filament dissolution takes place on applying voltage of opposite polarity [41]

To obtain a better understanding of the switching mechanism of metal ion-based CBRAM, let us consider an example of Ag/a-ZnO/Pt RRAM cells [41]. A general schematic illustration depicting the switching process of conductive bridge random access memory cell is shown in Fig. 5. The pristine state of the CBRAM memory cell is depicted in Fig. 5a. The Ag top electrode (TE) is an active component in the filament formation while the bottom Pt electrode is inert. On the application of the positive voltage bias to the Ag top electrode, the oxidation (Ag → Ag ^+^ + e ^−^) occurs at the top electrode because of which Ag ^+^ cations are generated and get deposited into the dielectric layer (a-ZnO) from the Ag electrode. The negative bias on the Pt bottom electrode (BE) attracts the Ag ^+^ cations, and as such, the reduction reaction (Ag ^+^ + e ^−^→ Ag) occurs at the bottom electrode. Thus, the Ag ^+^ cations are reduced to Ag atoms and accumulate until the conducting bridge is formed (Fig. 5b–d) and the RRAM device is said to exhibit LRS. This process is referred to as the ‘SET’. When the polarity of the applied voltage is reversed, the highly conducting filament dissolves almost completely and the device is said to be in the high resistance state (HRS). This process is referred to as ‘RESET’ and is depicted in Fig. 5e.

In oxygen vacancy-based RRAM (OxRRAM), the physical mechanism that is responsible for resistive switching is generally associated with the generation of oxygen vacancies (*V*$_{o}^{2+}$) and subsequent relocation of oxygen ions (O ^2−^), thus enabling the formation of conductive filament between the top and bottom electrodes of RRAM cell [59]. Initially, for the as fabricated RRAM cell, forming process, i.e. soft breakdown of the dielectric is essential. Once the soft dielectric breakdown occurs, the oxygen atoms are knocked out of the lattice on the application of high electric field towards the anode interface and become oxygen ions (O ^2−^) whereas the oxygen vacancies (*V*$_{o}^{2+}$) are left in the oxide layer. The oxygen ions (O ^2−^) react with anode materials or get discharged as neutral non-lattice oxygen, if the noble metals are used as materials for anode to form an interfacial oxide layer. Thus, the electrode/oxide interface behaves like an ‘oxygen reservoir’ [85]. Next, the accumulation of the oxygen vacancies (*V*$_{o}^{2+}$) in the bulk oxide switches the RRAM cell to the low resistance state (LRS) as the conductive filament (CF) is formed and the appreciable current flows in the device. To switch the device back to the high resistance state (HRS), the reset process occurs during which the oxygen ions (O ^2−^) migrate back to bulk oxide from anode interface and either combine with the oxygen vacancies (*V*$_{o}^{2+}$) or to oxidize the metallic CF precipitates and thus partially rupture the filament, thereby switching back the RRAM cell into HRS. For RRAM cells exhibiting unipolar switching mechanism, the diffusion of oxygen ions (O ^2−^) is activated thermally by Joule heating current and as such the oxygen ions diffuse from the interface or the region around the CF due to the concentration gradient. Also, it must be noted that a relatively higher reset current is required in unipolar switching RRAM to raise the local temperature around CF. In bipolar switching RRAM, on the other hand, the oxygen ions (O ^2−^) needs to be aided by the reverse electric field as the interfacial layer may present a significant diffusion barrier and pure thermal diffusion is not enough. It must be noted that the partial rupture of CF takes place in both the cases, switching the RRAM cell into the high resistance state (HRS). This is primarily due to the formation of oxygen vacancies (*V*$_{o}^{2+}$) and poor region resulting in the tunneling gap for electrons. To switch the device back to the LRS (SET process), the CF reconnects the electrodes as a result of the soft breakdown in the gap region. A similar set/reset process can repeat for many cycles.

Based on the above discussion, CBRAM also known as electrochemical metallization memory (ECM) relies on an electrochemically active metal electrode such as Ag, Cu, or Ni to form metal cation-based CF [95]. The CF in oxygen vacancies filament-based RRAM ‘OxRRAM’ also known as valence change memory (VCM) is composed of oxygen vacancy defects, instead of metal atoms, due to anion migration within the storage material itself [96]. Although the switching mechanism of both ‘OxRRAM’ and ‘CBRAM’ is discussed in detail, there is still some debate on the switching mechanisms of both the RRAM types [97]. For example, where the CF starts to grow in the set process and where to break in the reset process, and how these oxygen vacancies/metal atoms gather to form the CF. The current-voltage (I-V) characteristics of 20 consecutive switching cycles of Ta/TaO _*x*_/Pt [98]-based RRAM structure were investigated, and the clear variation of both LRS and HRS for consecutive switching cycles was observed. As a result, the overall memory window decreases, degrading the overall RRAM performance. This cycle-to-cycle resistance variability is primarily attributed to the random formation of CF as well as its rupture during the set and reset operation respectively.

A comparison of OxRRAM with CBRAM based on various operational parameters is shown in Table 3 [81, 84, 86–88, 99, 100]. This comparison reveals the striking difference in terms of the endurance characteristics of these RRAM memory types. This dissimilarity is because the conducting filaments of CBRAM are composed mainly of metal atoms which are relatively easier to drift and diffuse compared to oxygen vacancies, thus causing the degradation of the retention time and endurance characteristics of CBRAM compared to the OxRRAM. Although the switching mechanism of the both RRAM types are different, there are many common characteristics between the two of them. The only significant difference is that endurance for OxRRAM is significantly higher than of CBRAM.

**Table 3:** Comparison between metal-oxide RRAM and conductive bridge RRAM

Parameter	Metal-oxide RRAM	Conductive bridge RRAM
Speed (ns)	5 [81]	1 [99]
Operation voltage (V)	∼ 3 [51]	∼ 7 [86]
Operation current (*μ*A)	5 [51]	10 [87]
Endurance (cycles)	10^12^ [84]	10^6^ [41]
On/off ratio	10^7^ [88]	10^7^ [100]
Retention@ 85^∘^C (s)	10^6^ [44]	10^6^ [41]
Multilevel capacity	Yes	Yes
CMOS compatible	Yes	Yes
Fabrication	Easy	Easy
Scalability	Good	Good

## Performance Metrics of Resistive Random Access Memory (RRAM)

### Endurance

Resistive random access memory involves frequent transitions between a high resistance state (HRS) and low resistance state (LRS). Each switching event between the resistive states can introduce permanent damage and cause degradation of the RRAM performance. Endurance is thus defined as the number of times a RRAM device can be switched between the HRS and the LRS while ensuring a reliably distinguishable ratio between them [101]. Thus, an endurance test determines the maximum number of set/reset cycles that can be switched effectively before the HRS and the LRS are no longer distinguishable. The endurance characteristics of RRAM are obtained by performing a sequence of I-V sweeps in a resistive switching cell and the subsequent extraction of *R*_HRS_ and *R*_LRS_ on the application of a read voltage (typically 0.1 V) [41]. This method is reliable as one can obtain the correct switching of the device in each cycle; however, this method is very slow because the time required for obtaining an I-V sweep can be very higher particularly if the lower currents are involved.

**Fig. 6:**
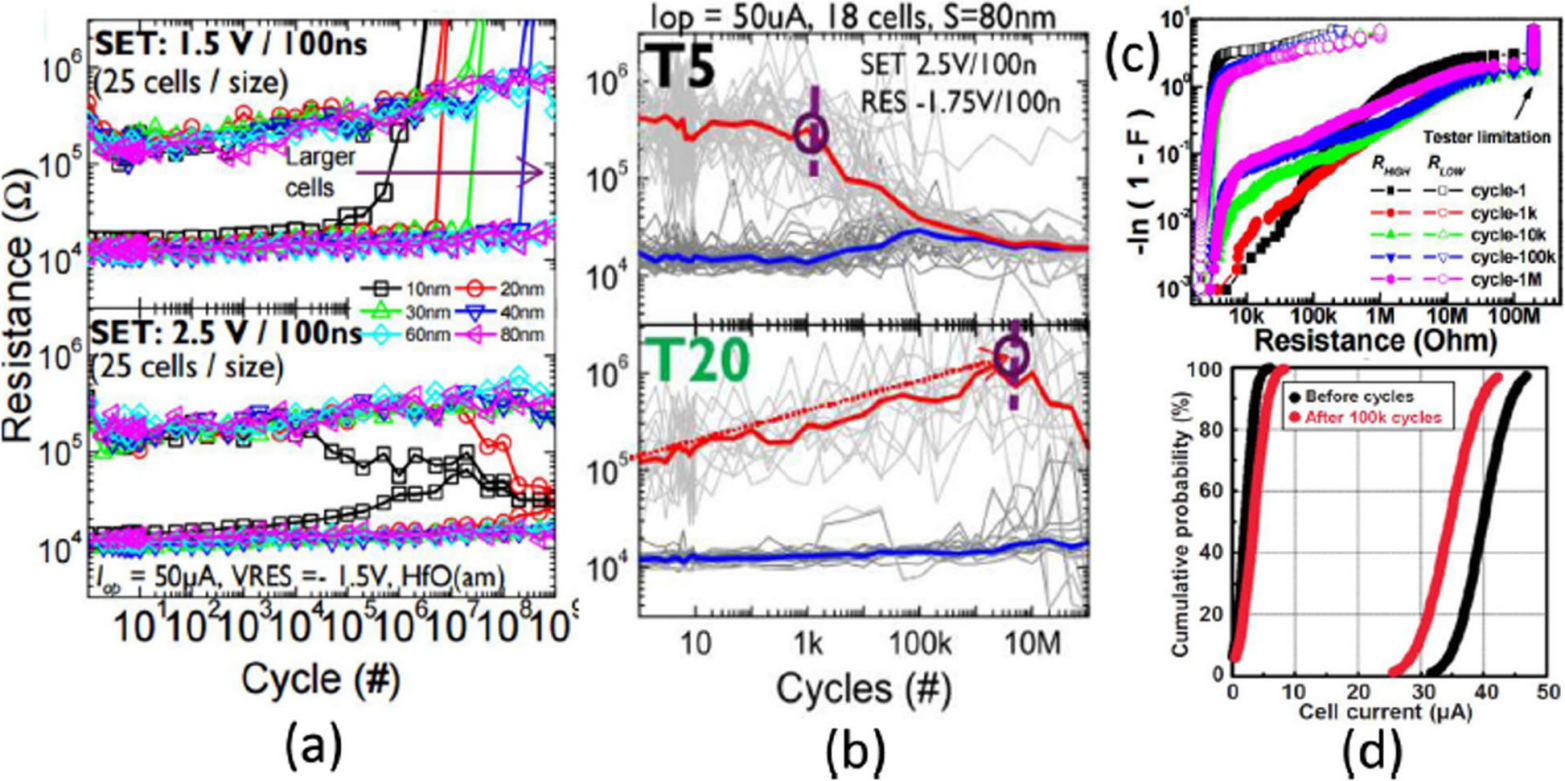
**a** Endurance cycles of *HfO*_*x*_-based RRAM at different SET voltage and cell size **b** with different thickness (T5= 2 nm, T20= 10 nm) at 2.5 V set voltage. **c** Resistance distribution of 1-kb array obtained from Weibull plots under different endurance cycles. **d** 100 k cycles endurance of 2-Mb-Ta_2_O_5_-based array; Reprinted from refs [102–104]

The endurance cycles in a *HfO*_*x*_ RRAM cell shows a strong dependence on the cell size, as is depicted in Fig. 6a, wherein better endurance in RRAM device with larger cell size is reported. In addition, vertically reducing the layer thickness results in degradation of endurance performance for SET voltage at 2.5 V as shown in Fig. 6b [102]. This degradation in endurance performance with downscaling of the switching layer is a result of the reduced number of ions in the active region. *HfO*_*x*_-based RRAM exhibits an excellent endurance performance of 10^6^ cycles on a 1-kb array with 30-nm cell size under 0.18 *μ*m technology and the same is shown in Fig. 6c [103]. By adding an extra layer of *AlO*_*x*_ above the bottom electrode (BE), array stability can be improved further as read disturb immunity for HRS is increased. For *TaO*_*x*_-based RRAM, a degradation in endurance performance with increasing pulse width and amplitude of RESET voltage was observed in Ta/Ta_2_O_5_/TiN RRAM structure [105]. A comparison of TiN and Ru bottom electrode in the Ta/Ta_2_O_5_/TiN RRAM shows that the main cause of endurance degradation is due to the reaction of oxygen ions with TiN electrode. Furthermore, an improved endurance of 10^9^ switching cycles was obtained without verification in a similar RRAM structure by reducing the Ta_2_O_5_ layer down to 3 nm [106] and use of triangular pulse having < 5 ns width. For large-scale array performance, a comparison of 2-Mb Ta_2_O_5_ memory before and after 10^5^ cycles of endurance test is shown in Fig. 6d [104]. The cell current distributions show a small variation for initial and final cycles. Also, the cell current for LRS falls below 50 *μ*A, indicating low power consumption of the array. The resistive switching devices with endurance higher than 10^12^ cycles have been reported in different types of RRAM cells involving tantalum oxide (TaO _*x*_)-based switching mediums [32, 36, 59]. Thus, tantalum oxide-based RRAM devices seem to be exhibiting the highest endurance.

### Retention

The data retention of a RRAM device involves investigating stability over a period of time for both LRS and HRS after undergoing set and reset transitions. In other words, the time period for which a memory cell will remain in a particular state after the set/reset operation determines the capability of a memory cell to retain its content [11]. The application of the constant voltage stress (CVS) over time using a low read voltage (0.1 V) and the measurement of the current versus time (I-t) curve for both LRS as well as the HRS enables the measurement of state retention. Due to the dispersing nature of atomic rearrangements induced in RRAM because of set voltage, the long retention time in LRS is difficult to obtain whereas, in HRS, retention is not a concern as it is usually the natural state of the device and RRAM will continue to remain in this state if no bias (or low bias) is applied. The retention in the LRS depends on the compliance limit during the SET transition, e.g. in RRAMs based on conductive filament switching mechanism, the larger compliance current produces a stronger conducting filament which is more stable over time [28, 41], as compared to a smaller compliance current. A projected endurance of 10 years at 85^∘^ C has been demonstrated in Ti/HfO_2_/TiN [18]. A commonly used method to obtain device endurance is by applying read pulse at high temperature after certain time intervals (e.g. every 1 s) and extrapolate the resistance to a 10-year period. Although this method is easy to implement, it has certain limitations primarily due to the read voltage stress applied to the cell. An alternative method is to change the temperature and record the time until the device fails. Activation energy is extracted by plotting the Arrhenius plot and extrapolate down to the operating temperature. However, the limitation of this method is that waiting is necessary until the failure occurs in the RRAM cell, and thus, this method is more time-consuming and expensive.

**Fig. 7:**
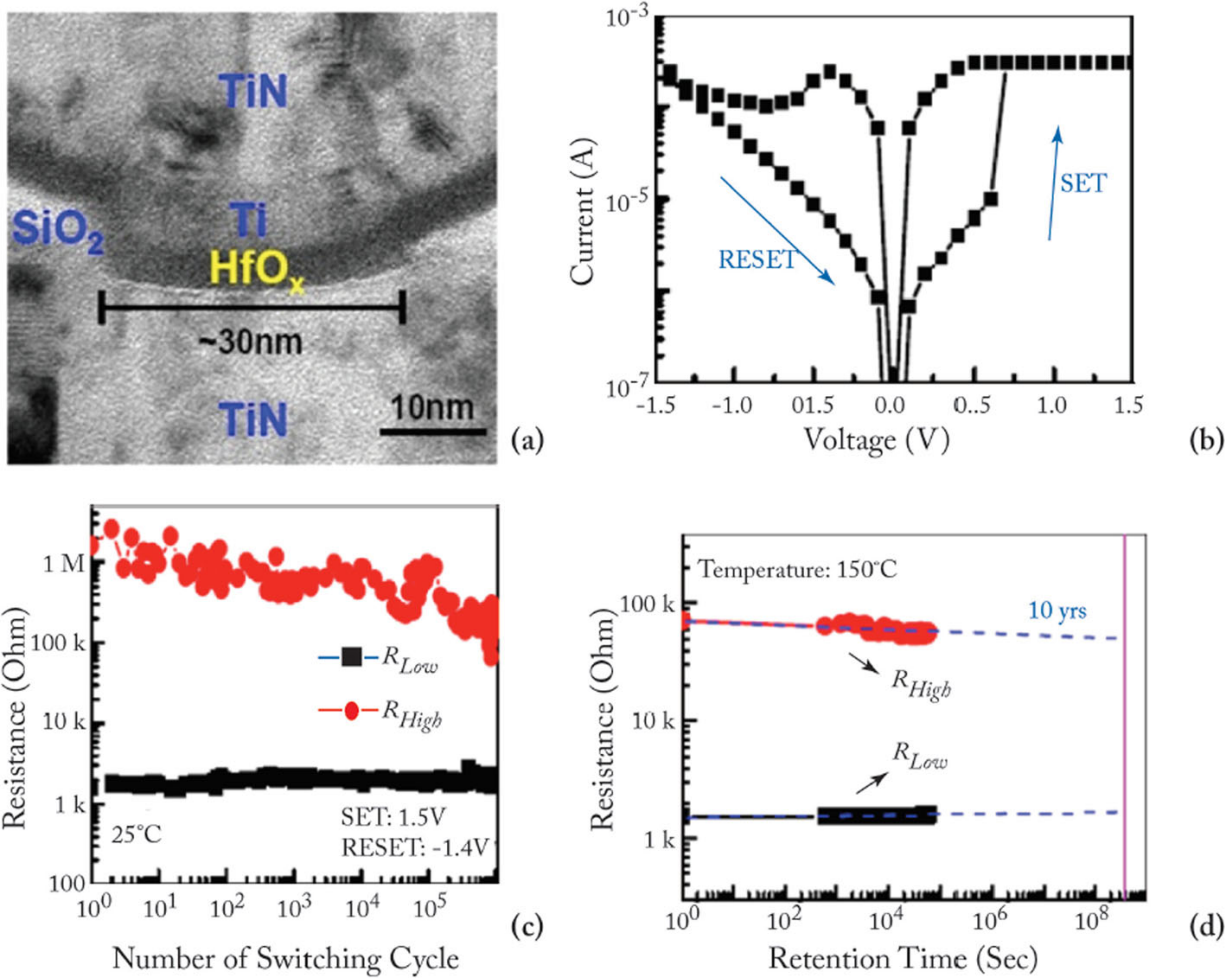
**a** Transmission electron microscopy (TEM) image of TiN/Ti/HfO _*x*_/TiN RRAM device. **b** Typical current-voltage (I-V) characteristics of the device with 30-nm cell size. **c** 10^6^ endurance switching cycles obtained from 500 *μ*s pulse. **d** A retention lifetime of 10 years is expected by testing at 150^∘^C ; reprinted from refs. [81, 103]

The device characteristics of *HfO*_*x*_-based RRAM [81, 103] developed at the Industrial Technology Research Institute, Taiwan, are demonstrated to further understand the working of RRAM device. The transmission electron microscopy (TEM) image of the TiN/Ti/ *HfO*_*x*_/TiN RRAM device with 30-nm cell size is shown in Fig. 7a. The device exhibits bipolar switching characteristics and the I-V curve obtained at 200 *μ*A set compliance current is shown in Fig. 7b. The device presents endurance of 10^6^ switching cycles with the resistance on/off ratio greater than 100 at set/reset programming conditions of + 1.5 V/– 1.4 V pulse with 500 *μ*s pulse width and the same is depicted in Fig. 7c.

### Uniformity

In RRAM cell, poor uniformity of various device characteristics is one of the significant factors limiting the manufacturing on a wider scale. The switching voltages, as well as both the HRS and the LRS resistances, are among the parameters exhibiting a high degree of variation. The variations of the resistance switching include temporal fluctuations (cycle-to-cycle) and spatial fluctuations (device-to-device). The stochastic nature of the formation and rupture of conductive filament is believed to be the main reason for these variations. Cycle-to-cycle and device-to-device variability is a major hindrance for information storage in RRAM devices [59]. The observation of cycle-to-cycle variability is influenced by the change in the number of oxygen vacancy defects that arise in the CF due to its stochastic nature of formation and rupture during the switching event [107]. Due to this random nature of the CF, the prediction and the precise control of the shape of the CF becomes extremely challenging. This variability becomes worse as the compliance limit (i.e. compliance current ‘*I*_cc_’) is reduced. On the other hand, for the higher value of ‘*I*_cc_’, the ratio of standard deviation (*σ*) and average resistance (*μ*) is low, resulting in a smaller LRS resistance spread. This is attributed to the higher defects in the CF, thus forming a well-defined path for current conduction.

**Fig. 8:**
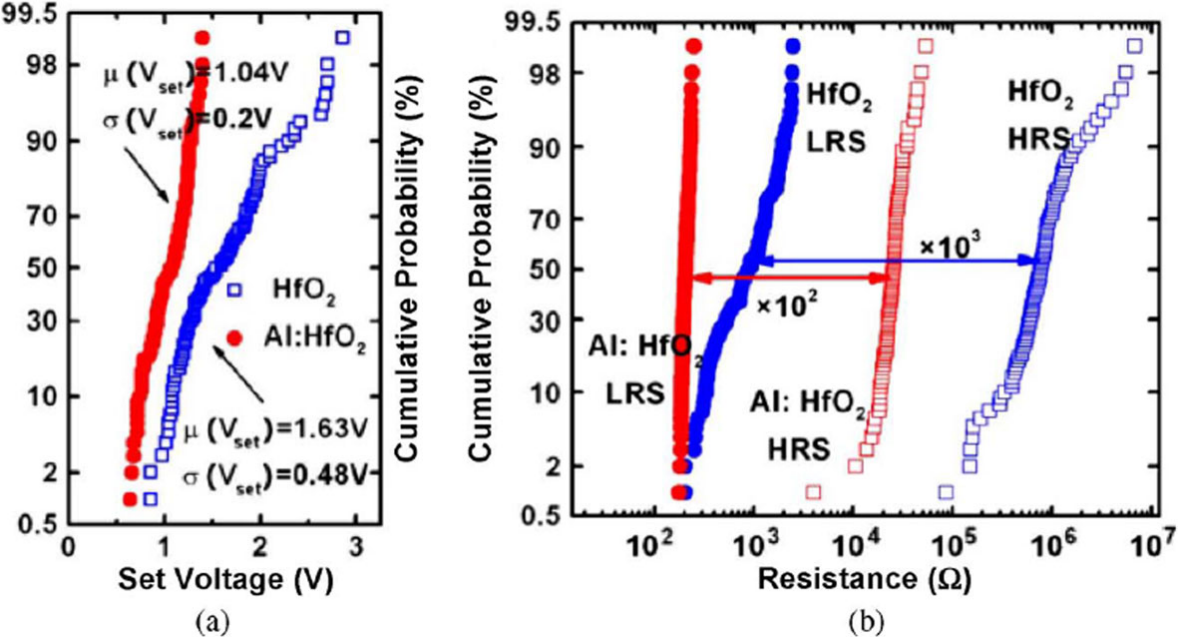
Uniformity improvement of Al buffered HfO _*x*_ RRAM compared to HfO _*x*_-based RRAM array. **a** Statistical distribution of SET voltage (*V*_set_) obtained from 100 DC sweep cycles. **b** HRS and LRS statistical distribution for 100 pulse sweep cycles; reprinted from ref. [59]

RRAM also exhibits device-to-device (cell-to-cell) non-uniformity which also degrades the memory performance by reducing the memory margin between two states. The origin of this variability is attributed to the non-uniformities in the fabrication process such as the thickness of the switching film, etching damages and surface roughness of the electrodes. A lot of research has been conducted to improve the uniformity of RRAM and several methods have been explored for the same. One of the methods utilizes the concept of inserting nano-crystal seeds which confine the paths of the conductive filament by enhancing the effect of local electric field [82, 90, 108]. In Ti/TiO _2−*x*_ /Au-based RRAM [28], the induction of platinum (Pt) nano-crystals within the thin TiO _2−*x*_ results in an enhanced uniformity of the RRAM cell. The Pt nano-crystals limit the switching effect into regions with high oxygen vacancy generation probability which results in improved uniformity. In another approach, engineering the electrode/oxide interface by embedding appropriate buffer layers is very useful in achieving uniform RRAM operation. In HfO _*x*_-based RRAM [109], a thin Al buffer layer is inserted between the TiN electrode and HfO _*x*_ oxide layer. This results in significant improvement of set voltage distribution as well as the resistance distribution, thus enhancing the uniformity of the device. The improvement in the SET voltage and the resistance distribution of the RRAM device after inserting a thin Al buffer layer between TiN electrode and HfO _*x*_ bulk oxide and the same is depicted in Fig. 8 [59]. Al atoms are assumed to diffuse into HfO_2_ thin films, and they tend to localize oxygen vacancies due to the reduced oxygen vacancy formation energy, thus stabilizing the generation of conductive filaments, which helps to improve the resistance switching uniformity.

In addition to the materials engineering approach, a novel programming method has also been suggested to reduce fluctuations. A multistep forming technique was implemented in W/HfO_2_/Zr/TiN [22]-based RRAM to minimize the overshoot current due to the parasitic effects. A multi-step forming technique results in the gradual formation of the filament; thus, a low set/reset current is achieved improving the switching characteristics of the device. Various other methods such as constant voltage forming and hot forming (usually referred to as forming at a higher temperature) have also been investigated to effectively reduce the resistance variations [110]. Another method of achieving high uniformity is by applying a pulse train rather than a single pulse to a RRAM cell [23]. This approach not only results in improved uniformity but also enhances the multilevel capability of a RRAM cell.

### Effect of Operating Temperature and Random Telegraph Noise

To achieve a reliable performance of the RRAM device, the effect of operating temperature and random telegraph noise (RTN) is investigated. It is observed that the resistance of both the LRS and HRS states undergoes variations because of the change of operating temperature. The temperature study of TiN/HfO_2_/Ti/TiN [111] was carried out. A positive sweep voltage of < 3 V magnitude and compliance current of 1 *μ*A was applied for the electroforming. Once forming takes place, a reset voltage (*V*_reset_) < –1 V switches the device back to the HRS (OFF state). To switch the device back to the LRS (ON state), set voltage (*V*_set_) <1 V is applied.

**Fig. 9:**
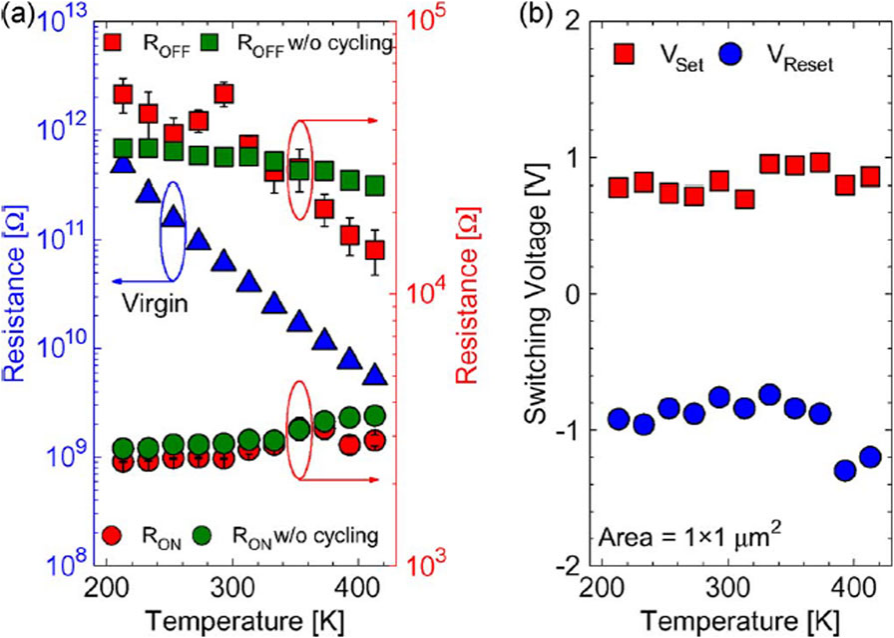
The effect of varying temperature on **a** virgin resistance (left axis) and the OFF-state as well as the ON-state resistances (right axis) at 213–413K temperature range and **b** switching voltages *V*_set_ and *V*_reset_ ; reprinted from ref. [111]

The reset operation in RRAM device tends to show voltage-controlled negative differential resistance (NDR). The reset operation occurs abruptly at low temperatures, while for temperatures above room temperature, the reset process takes place more gradually. The resistance of the RRAM device in the pristine state, as well as the ON state and OFF state as a function of temperature is depicted in Fig. 9a. The semiconducting behavior is observed for the pristine state as well as the OFF state, i.e. resistance decreases with increase of temperature. For the ON state, a metallic characteristic is observed, i.e. resistance increases with increase of temperature. Due to the variation of resistance with change in temperature, R _OFF_/R _ON_ also decreases from a value of 20 to approximately 5 over the temperature range of 213–413K. In Ti/HfO _*x*_/Pt devices, decrease in R _OFF_/R _ON_ was observed with temperature-dependent cycling. This decrease in resistance ratio was attributed to the built-up of oxygen-vacancy-related traps inside the HfO_2_ metal oxide layer [112, 113]. Additionally, temperature-dependent measurements without set/reset operation were carried out to evaluate the impact of I-V cycling on the R _OFF_/R _ON_ ratio. The sweep voltage across the RRAM device was stopped before reaching *V*_set_ and *V*_reset_ values. For OFF state resistance (green rectangles), a weaker temperature dependence was observed in contrast to the ON state resistance (green circles) which exhibited similar characteristics, compared to the cycling case. From these observations, we infer that I-V cycling induces stronger temperature dependence, which decreases the *R*_OFF_/*R*_ON_ ratio. The effect of temperature variation on the switching voltages *V*_set_ and *V*_reset_ is depicted in Fig. 9b. The slight variation in *V*_set_ with changing temperature indicates that there is no significant temperature difference. For the case of *V*_reset_, the general trend is that a decrease in voltage value of about 0.2 V with temperature increase in the range of 233–333K is observed. Also, a slow increase of *V*_reset_ is observed for 353–413K temperature range.

**Fig. 10:**
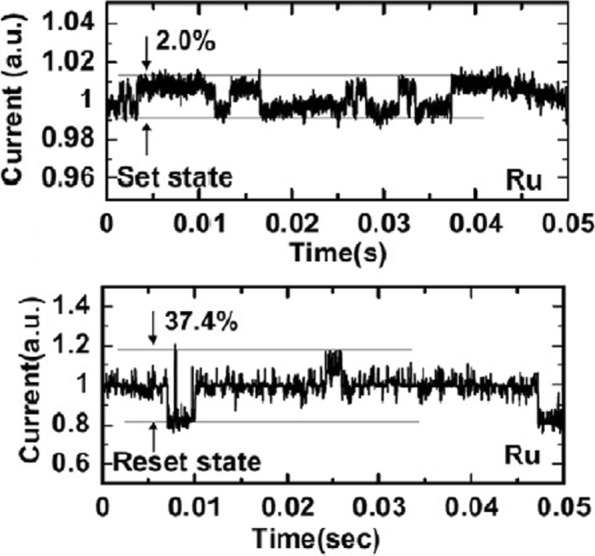
Complex RTN signals in LRS and HRS of Ta_2_O_5_/TiO_2_ -based RRAM depicting normalized noise amplitude and average current; reprinted from ref. [114]

Random telegraph noise (RTN) is another factor that affects the performance of RRAM. RTN has for long been used as an indicator of device performance and reliability. RTN decreases the memory margin between the HRS and LRS because of the extensive fluctuations in the read current during the read operation. Due to the effect of RTN, the read margin, scaling potential and the multilevel cell capability of a RRAM cell are greatly affected [114]; thus, it needs to be investigated to achieve reliable performance. To investigate the effect of bottom electrode on RTN, an analysis of Ta_2_O_5_/TiO_2_ RRAM [115] was carried out. The examples of complex RTN signals in LRS and HRS are depicted in Fig. 10. RTN causes read instability in the RRAM device, thus reducing the read margin, multibit storage implementation and hindering device scaling. The RTN is attributed to the trapping and de-trapping of electrons in the proximity of the CF in LRS whereas it occurs in the tunneling gap in the HRS state. Although the physics of RTN is still not clear and is being highly debated, the electron trapping and de-trapping which temporarily inhibits the charge transport is widely accepted as the mechanism responsible for fluctuation due to RTN. It is observed that with the decrease in operation current, the amplitude of RTN increases, thus highly affecting the HRS level. Therefore, it is necessary to ensure the additional resistance margin to achieve reliable performance. RTN in RRAM has been researched extensively, although the physical mechanism of RTN is still not explicitly defined. RTN can be utilized as a tool to map the movements of active vacancies in RRAM due to its time-dependent variation. This might be quite useful to understand the failure mechanisms of other reliability issues.

## Multilevel Resistive Random Access Memory (RRAM)

### Multilevel Per Cell (mlc) Storage

Owing to their small physical size and low power consumption, RRAM devices are potential for future memory and logic applications. Increased storage density is among the most critical aspects of memory technology to enable the design of multibit capacity [89] memory cells. The multiple resistive states can be achieved in RRAM cells which provide benefits of low-cost and high-density non-volatile data storage solutions. Currently, a lot of research is being conducted in the area of RRAM to scale down the dimensions and increase the structural density of memory arrays. Previously, the storage density of RRAM has been increased by the reduction of device size; however, the complexity involved in the experimental procedures limits its successful implementation. Another suggested method is employing three-dimensional (3D) crossbar architectures. Two types of architectures of ‘vertical’ and ‘crossbar RRAM’ have been proposed [116, 117]; however, both these architecture types require advanced fabrication procedures which is not desirable. A much simpler alternative to increase storage density in RRAM devices is by making use of multilevel cell (MLC) storage technology which enables storing more than one bit per cell without reducing the physical device dimensions. This MLC is one of the most promising properties of RRAM which can significantly increase the memory storage density [83, 118–125]. Thus, instead of a single high and low resistance state (HRS and LRS), we can achieve multiple HRS and LRS, without changing the device dimensions. However, to achieve reliable MLC operation, the precise control over the resistance of the different resistance levels of RRAM should be ensured; otherwise, the device will suffer from resistance variability and reliability issues mainly due to the random nature of the conductive filament formation during the switching process [126].

### Methods to Obtain Multilevel Per Cell (mlc) Modes in RRAM

The MLC behavior in RRAM makes it very useful for high-density applications. To obtain MLC behavior in RRAM, the following three methods are employed: (i) changing compliance current, (ii) controlling reset voltage and (iii) varying pulse width of program/erase operation.

#### MLC by Changing Compliance Current

**Fig. 11:**
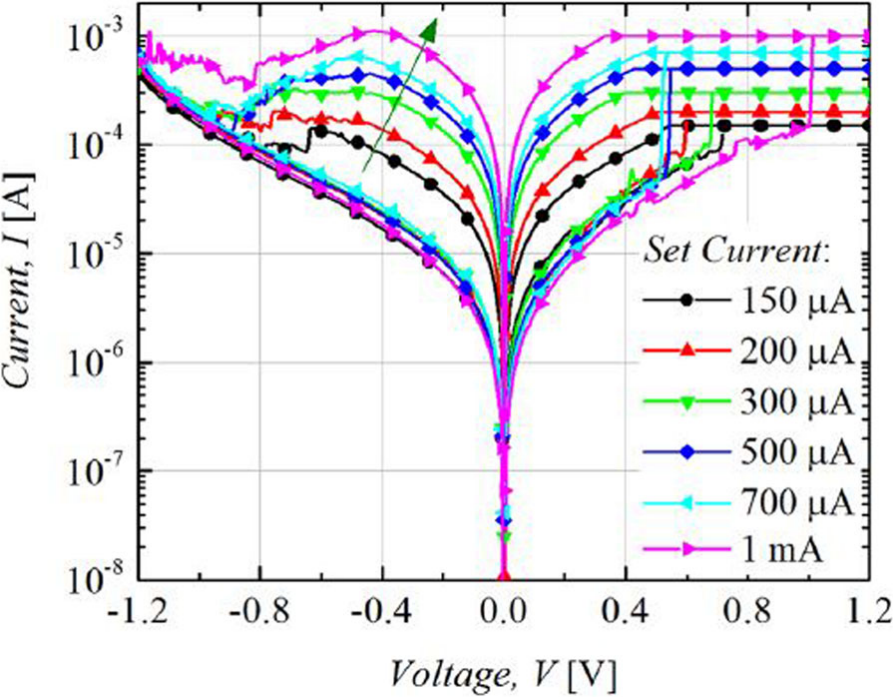
Multilevel characteristics of Ti/Ta_2_O_5_/Pt RRAM obtained by controlling the compliance current. ‘Reproduced from [127], with the permission of AIP Publishing’

The RRAM device is usually operated with 1-RRAM (1R) cell configuration [41] or in 1-Transistor 1-RRAM (1T-1R) cell configuration [18]. The MLC characteristics in 1R configuration can be obtained by changing the current compliance (*I*_cc_) during ‘set’ operation whereas the MLC characteristics in 1-Transistor 1-RRAM (1T-1R) cell structure are controlled by varying the voltage at the gate of the transistor, which enables the control of compliance current (I _*cc*_) during the set operation of a RRAM cell. The typical MLC I-V curves of Ti/Ta_2_O_5_/Pt [127] based RRAM cell are shown in Fig. 11. As the compliance current (*I*_cc_) is increased from 150 *μ* A to 1 mA, six different LRS are obtained at *I*_cc_ = 150 *μ*A, *I*_cc_ = 200 *μ*A, *I*_cc_ = 300 *μ*A, *I*_cc_ = 500 *μ*A and *I*_cc_ = 700 *μ*A, *I*_cc_ = 1 mA due to the increase in the respective current of LRS (I _*LRS*_) while the HRS is maintained constant and the HRS current (I _HRS_) remains same for all the LRS levels. For Ti/Ta_2_O_5_/Pt RRAM, with the increase in *I*_cc_, the maximum reset current (I _reset_) also increases while the set voltage is almost maintained constant. Also, it was observed that the resistance of the LRS (R _LRS_) decreases while the (I _reset_) increases owing to the stronger filament formation with the increase in *I*_cc_.

**Fig. 12:**
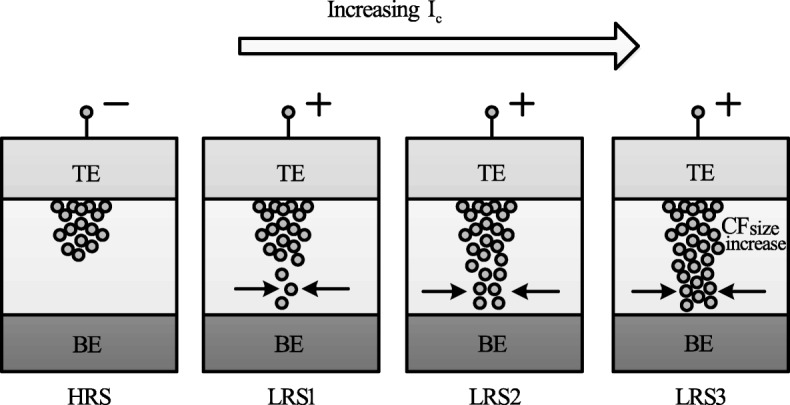
Schematic illustration of multiple resistance states in RRAM cell obtained by varying compliance current ‘*I*_cc_’ [98]

The formation of the CF and its corresponding widening with an increase in *I*_cc_ is the attributed mechanism of multilevel per cell (MLC) in compliance current (*I*_cc_) mode as depicted schematically in Fig. 12. With an increase in the size of CF because of an increase of *I*_cc_, the resistance of the CF decreases and hence results in multiple LRS levels for different values of *I*_cc_. It is also observed that I _reset_ increases with increasing *I*_cc_ as higher power is required to rupture the CF having larger diameter.

#### MLC by Controlling Reset Voltage

**Fig. 13:**
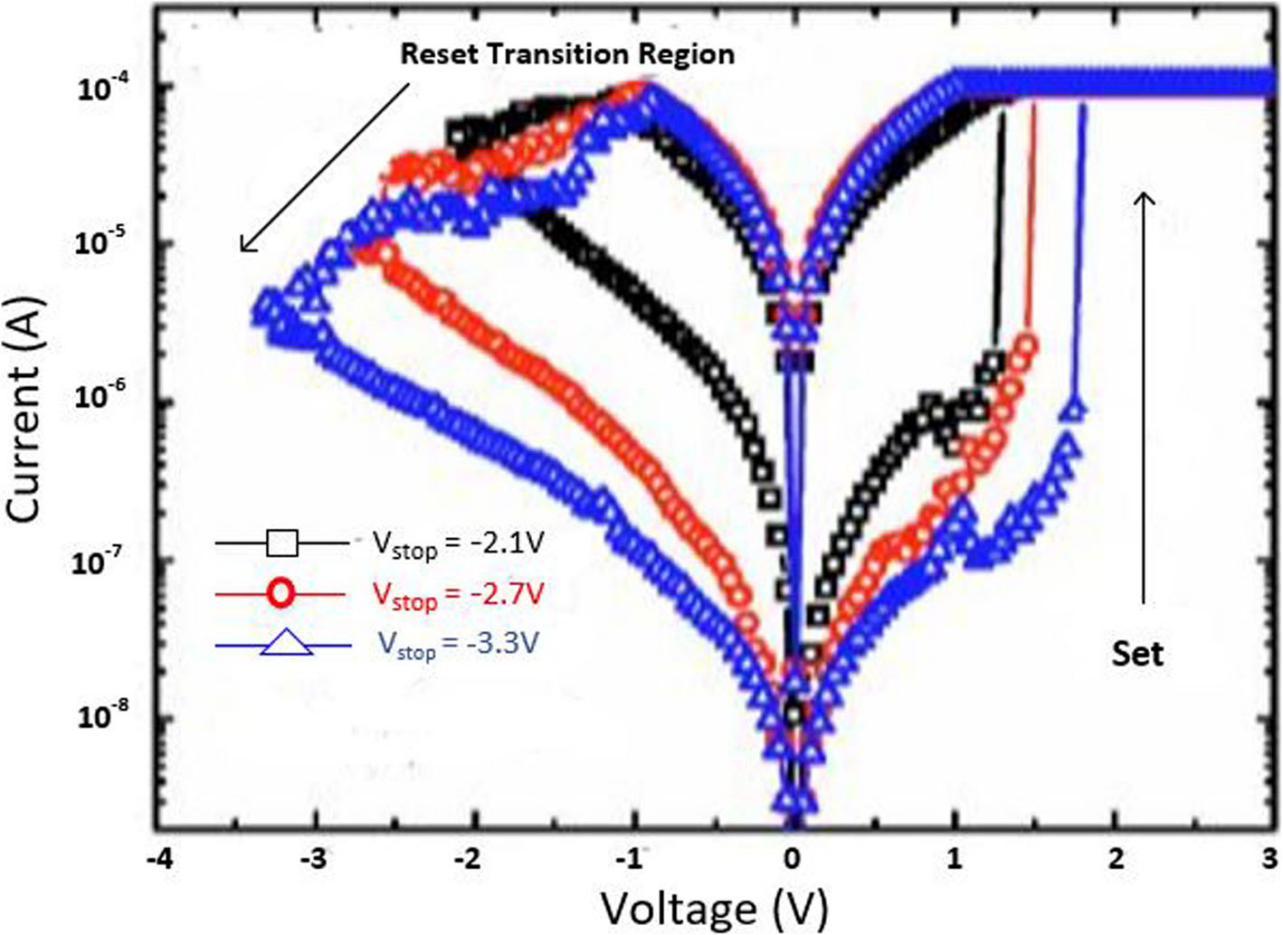
Multilevel characteristics of TiN/HfO _*x*_/AlO _*x*_/Pt RRAM obtained by controlling the reset voltage. ‘Reproduced from [128], with the permission of AIP Publishing’

The MLC characteristics in a RRAM cell can also be obtained by controlling the reset voltage (*V*_reset_) while (*I*_cc_) is maintained constant. In this case, the typical MLC I-V curves of TiN/HfO _*x*_/AlO _*x*_/Pt-based RRAM cell [128] by applying different (*V*_reset_) of − 2.1 V, − 2.7 V and − 3.3 V are shown in Fig. 13.

It is observed that with an increase in (*V*_reset_), the HRS current (*I*_HRS_) decreases; thus, multiple HRS levels with the same LRS resistance are obtained. In addition, the set voltage (*V*_set_) also increases as *V*_reset_ is increased while as the *I*_reset_ remains almost constant.

**Fig. 14:**
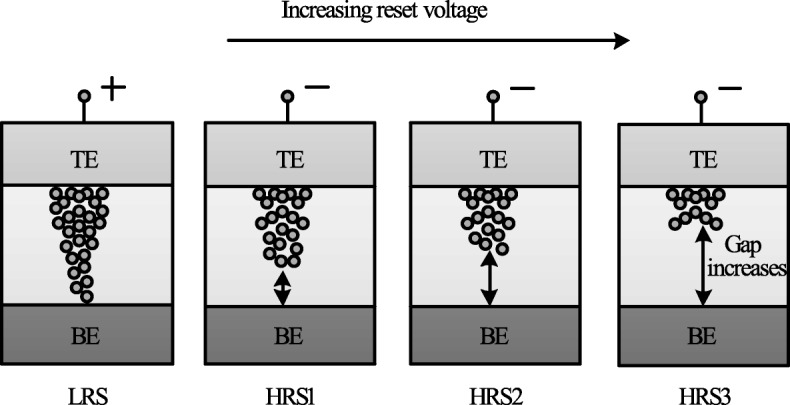
Schematic illustration of multiple resistance states in RRAM cell obtained by varying reset voltage ‘V _reset_’ [98]

The decrease in *I*_HRS_ with the increase in reset voltage is primarily due to the increase in the gap between the metal electrode and tip of the CF as depicted in Fig. 14. The more the magnitude of the *V*_reset_, the larger the gap and thus the higher the value of resistance. Therefore, an increase in the gap between the CF tip and bottom electrode (BE) with increasing reset voltage results in multiple resistance levels of HRS. It is observed that the devices in which the *I*_reset_ shows a gradual change in current instead of the abrupt change during the ‘reset’ operation, the change in HRS resistance in such devices can be due to decrease in the size of the conductive filament (CF) as *V*_reset_ is increased. This approach is more viable practically for cross-point architectures as it requires relatively lower complex circuitry.

#### MLC by Varying Program/erase Pulse Width

MLC characteristics can also be obtained by varying the program/erase pulse width while the amplitude of the pulse is maintained constant [23]. In HfO _*x*_-based RRAM [128], three HRS levels were demonstrated by varying the width of the reset pulse from 50 ns to 5 *μ*s. This method of obtaining MLC characteristics in RRAM is relatively easier; however, this scheme is energy inefficient. This drawback limits the application of this method to obtain reliable characteristics in the RRAM cell. The higher energy consumption of the RRAM device was confirmed on the comparison of the transient responses between the reset pulse amplitude and pulse width control. This is particularly due to the higher unwanted energy dissipation as the thermal energy in the resistive switching material.

A summary of RRAM devices exhibiting multiple resistance states is shown in Table 4. As is evident from the table, various RRAM devices with multiple resistance states have been reported. Till date, however, only 8 resistance states have been demonstrated in a single RRAM cell either by varying *I*_cc_ or *V*_reset_. Therefore, there is a huge scope for increasing the number of resistance states in the RRAM cell, thus enhancing its storage density.

**Table 4:** RRAM devices exhibiting multiple resistance states

Reference	RRAM device	Resistance states	Method used
[22]	W/ HfO_2_/ Zr/ TiN	4 states: 1HRS, 3LRS	Varying *V*_reset_
[23]	TiN/ HfO_2_/ Pt	8 states: 7HRS, 1LRS	Varying *V*_reset_
[27]	Ti/ TiN/ TiO _2−*x*_/ Au	6 states: 1HRS, 5LRS	Varying *I*_cc_
[29]	Ti/ TiO _2−*x*_/ Au	7 states: 1HRS, 6LRS	Varying *I*_cc_
[32]	Pt/ TaO _*x*_/ TiN	4 states: 1HRS, 3LRS	Varying *I*_cc_
[36]	W/ Ta/ TaO _*x*_/ Pt	8 states: 1HRS, 7LRS	Varying *I*_cc_
[41]	Ag/ a-Zno/ Pt	6 states: 1HRS, 5LRS	Varying *I*_cc_
[43]	Au/ Zno/ ITO	4 states: 1HRS, 3LRS	Varying *I*_cc_
[47]	ITO/ Zn_2_TiO_4_/ Pt	4 states: 1HRS, 3LRS	Varying *I*_cc_
[89]	Au/Ti/ TiO _2−*x*_/Au	6 states: 1HRS, 5LRS	Varying *I*_cc_
[83]	TiN/ HfO _*x*_/AlO _*x*_/ Pt	4 states: 3HRS, 1LRS	Varying *V*_reset_
[122]	Ru/ Ta_2_O_5_/TiO_2_/ Ru	4 states: 3HRS, 1LRS	Varying *V*_reset_
[124]	Pt/W/ TaO _*x*_/ Pt	8 states: 7HRS, 1LRS	Varying *V*_reset_

## Modeling of RRAM Devices

Modeling plays a very critical role in development of devices utilizing semiconductor technologies. To fully understand device operation and to optimize the performance, an accurate model is of great importance. A number of RRAM models with varying features and accuracy have been proposed [129]. This section discusses the characteristics and attributes of the various commonly used RRAM models popular.

### Stanford/ASU Model

One of the most popular physics-based RRAM models is the Stanford/ASU RRAM model [130–132], proposed by Guan et al. and Chen et al. This model was applied to validate the I-V switching characteristics of HfO_2_ RRAM [128] and includes the effect of Joule heating and temperature change on the switching of RRAM devices.

This model is dependent on the CF growth inside a dielectric switching layer. The filament gap, i.e. the gap between the tip of the CF and top electrode, is the internal state variable for this model. The growth of CF inside a dielectric is attributed to the oxygen ion movement and regeneration and recombination of oxygen vacancies [133]. Thus, the rate of change of filament gap (g) is given as [130]:

1 $$ {\frac{dg}{dt}} = V_{\tiny{0}}.\exp\bigg({{\frac{-E_{a},m}{k_{b}.T}} }\bigg). {\text{Sinh}} \bigg({\frac{qa_{h}\gamma V}{L.k_{b}.T}}\bigg)  $$

where *E*_*a*_ is the activation energy, *V*is the magnitude of the voltage applied across the device, *L* is the switching material thickness, *a*_*h*_ is the hopping distance, *γ* is the local field enhancement factor, *V*_0_ is the velocity containing attempt to escape frequency, *K*_*b*_ is the Boltzmann constant, *q* is the elementary unit charge and *T* is the temperature of the conductive filament.

The spatial variation in the gap size is accounted for in this model, in addition to the variations which arise due to the stochastic property of the ion process. A noise signal is added to the gap distance to account for these variations as [130]:

2 $$ g_{|t+\Delta t} = F \Big[ g_{|t}, {\frac{dg}{dt}} \Big] + \delta_{g}\times\tilde{X}(n)\Delta t, n = {\frac{t}{T_{GN}}}  $$

where *Δ*t is the simulation time step, the function *F* represents time evolution of gap size from*t* to *Δ*t. $\tilde {X}$(n) is a zero mean Gaussian noise sequence. *T*_GN_ is the time interval after which $\tilde {X}$(*n*) changes to next random value.

The variation in the gap size *δ*_*g*_ depends on kinetic energy of ions and filament temperature as [130]:

3 $$ \delta_{g} (T) = {\frac{\delta^{\tiny{0}}_{g}}{\bigg\{ 1+\exp \Big({\frac{T_{\text{crit}}- T}{T_{\text{smith}}}} \Big) \bigg\}}}  $$

where $\delta ^{0}_{g}$ and *T*_smith_ are fitting coefficients to match the resistance distribution curves to experiments and *T*_crit_ is a threshold temperature above which the gap size changes significantly.

This model shows strong dependence on temperature; thus, there is a need to account for the change of *‘T’*. With change in cell characteristics, the dynamic inner domain temperature *T* changes significantly, while the outer domain assumed to be at uniform and stable temperature (T _bath_), is related as [130]:

4 $$ c_{p} {\frac{dT}{dt}} = V(t).I(t) - k(T-T_{\text{bath}})  $$

where C _*p*_ is the effective heat capacitance of inner domain, *V*(t) *I*(t) represents the Joule heating and *k*is the effective thermal conductivity.

Using a generalized conduction mechanism, the current conduction is defined as [130]:

5 $$ I(g,v) = I_{\tiny{0}}.\exp\bigg({{\frac{-g}{g_{\tiny{0}}}} }\bigg){\text{Sinh}} \bigg({\frac{V}{V_{\tiny{0}}}}\bigg)  $$

where *I*_0_, *g*_0_ and *V*_0_ are the fitting parameters to match experimental results.

One of the significant features of this model is its implementation in neuromorphic applications and RRAM synaptic device design [134], giving the model a great degree of flexibility and further scope for implementation in various neuromorphic systems.

### Physical Electro-thermal Model

Physical electro-thermal model was developed by Kim et al. [135] and implemented with tantalum pentoxide (Ta_2_O_5_) -based bilayer RRAM [136–138]. This physical model solves the differential equations based on finite element solving method. This model also makes use of electrothermal physics phenomenon approach for modeling [139], thus giving it advantage in terms of flexibility to incorporate finite element method (FEM) solver to simulate the system very accurately. However, the drawback of this approach is its difficulty in implementation for SPICE and Verilog circuit solvers.

This model describes CF as a doped region having oxygen vacancies as dopants with CF extending from the top to the bottom electrode of the device. To describe the drift-diffusion of vacancy migration, this model assumes the same equation can be used to describe both the processes of oxygen ions and vacancies. The ion model by Mott and Gurney [140] is employed here to describe the process given as [135]:

6 $$ {\frac{dn_{D}}{dt}} = \Delta \times \bigg(D_{s}.\Delta_{n\tiny{D}}- \mu v n_{D} \bigg) + G  $$

where *D*_*s*_ describes the diffusion process, *v* gives the drift velocity of vacancies and *G* is the CF growth rate which actually describes the SET process. The parameters are defined as [135]:

7 $$ D_{s} = {\frac{1}{2}} \times a^{2} \times f_{e} \times \exp \bigg({\frac{- E_{a}}{k_{B}T}} \bigg)  $$

8 $$ v = a_{h} \times f \times \exp \bigg({\frac{- E_{a}}{k_{B}T}} \bigg) \times {\text{Sinh}} \bigg({\frac{q a_{h}E}{k_{B}T}} \bigg)  $$

9 $$ G = A \times \exp \bigg({\frac{- (E_{a}-ql_{m}E)}{k_{B}T}} \bigg)  $$

where l _*m*_ is the mesh size.

These equations govern the physical transformation of the device during SET and RESET transition, thus essentially controlling the CF growth and rupture.

### Huang’s Physical Model

Huang’s physical model developed by Huang et al. [141, 142] is a very comprehensive physical model for RRAM device as it takes into account both the CF width and the gap of filament to electrode as the factors affecting the state variable dynamics. In addition, temperature distribution is also accounted for in this model.

SET/RESET process is considered as a result of generation/recombination process of oxygen ions (*O*^2−^) and oxygen vacancies (*V*_0_). During the SET process, CF starts to evolve from the tip of the top electrode (T.E) and elongates in radius with increase in voltage, resulting in final width ‘W’ of the C.F. This model assumes symmetrical cylindrical shape of the C.F. During RESET process, CF ruptures starting from TE till it dissolves completely with increase in voltage. The filament gap distance ‘x’ is defined as the gap between active electrode layer (T.E) and the tip of the C.F.

Thus, for the SET process, parameter ‘W’ acts as state variable, while for RESET, parameter ‘x’ acts as state variable. Therefore, $\frac {dx}{dt}$ and $\frac {dw}{dt}$ define the dynamics of the device during the SET/RESET transition.

During the first reset process, CF reduction rate, i.e. release of *O*^2−^, is by the electrode is expressed as [142]:

10 $$ {\frac{dx}{dt}} = a \times f\times \exp \bigg({\frac{- E_{i}-\gamma Z_{e}V}{k_{B}T}} \bigg)  $$

For *O*^2−^ hopping within the oxide layer, the CF reduction rate with ‘a’ being the distance between two V_0_ is given as [142]:

11 $$ {\frac{dx}{dt}} = a \times f\times \exp \bigg({\frac{- E_{h}}{k_{B}T}} \bigg) {\text{Sinh}} \bigg({\frac{ a_{h}Z_{e}E}{k_{B}T}} \bigg)  $$

For the case of RESET process when dominated by recombination between *O*^2−^ and V_0_ is expressed as [142]:

12 $$ {\frac{dx}{dt}} = a \times f\times \exp \bigg({\frac{- \Delta E_{r}}{k_{B}T}} \bigg)  $$

In the initial step of the SET process dominated by recombination of oxygen vacancies with thin CF initially grown is given by [142]:

13 $$ {\frac{dx}{dt}} = -a \times f_{e}\times \exp \bigg({\frac{- E_{a}-\alpha_{a} Z_{e}E}{k_{B}T}} \bigg)  $$

Here, *Z* and *α*_*g*_ are the fitting parameters.

For the second step, CF grows along its radial direction and is defined as [142]:

14 $$ {\frac{dw}{dt}} = \bigg(\Delta w + {\frac{\Delta w^{2}}{2w}} \bigg) \times f_{e}\times \exp \bigg({\frac{- E_{a}-\gamma Z_{e}v}{k_{B}T}} \bigg)  $$

The current flowing through the device is modeled as a correlation of hopping current with voltage and gap distance expressed by [134] as:

15 $$ i = i_{0}. \exp \bigg({\frac{-x}{x_{T}}} \bigg) {\text{Sinh}} \bigg({\frac{v}{v_{T}}} \bigg)  $$

This model is validated in HfO _*x*_/TiO _*x*_ system [141, 142], and a pretty accurate match between the experimental and simulation results is obtained. Although this model accounts for the significant processes which affect the RRAM operation, however, it has some limitations. The most critical one is being incompatible with the SPICE and Verilog-A.

### Filament Dissolution Model

This model was developed exclusively for unipolar RRAM devices by Russo et al. [143–145], however was later modified for bipolar RRAM devices [139, 146] also. Filament dissolution model is based on rupture of CF under the effect of significant temperature change caused due to Joule heating.

One of the significant advantages of this model is that it utilizes the simple partial differential equations to account for the device current and temperature changes due to Joule heating as well as the dissolution velocity. The conduction of current within the device is described by Poisson’s equation [144] as:

16 $$ \triangledown \times \bigg({\frac{1}{\varphi}\triangledown_{v}} \bigg) = 0  $$

Here, *φ* is the oxide resistivity and *v*defines the electric potential due to the application of external bias voltage to one of the electrodes while the other electrode is connected to ground.

The CF is divided into a number of mesh grids and at each point of the mesh grid the temperature is calculated to describe the rupture of CF. The Fourier steady-state heat equation describes this effect as [144]:

17 $$ -\triangledown \times \bigg(k \triangledown T \bigg) = \varphi J^{2}  $$

where *k*represents the oxide layer thermal conductivity, *J*is the current density and *T*is the device temperature.

The temperature ‘T’ of the device increases to the critical temperature, after which the device is reset and the CF dissolution takes place. The dissolution factor is modeled as [144]:

18 $$ V_{\text{DIS}} = V_{\text{DIS}-F}. \exp \bigg({\frac{- E_{a}}{k_{B}T}} \bigg)  $$

where *E*_*a*_ is the activation energy, *k*_*b*_ is the Boltzmann constant, *V*_DIS−*F*_ is a fitting parameter and *V*_DIS_ is velocity of CF boundary towards symmetry axis.

The resistivity of CF is temperature-dependent and is described as [144]:

19 $$ \varphi_{\text{CF}} (T) = \varphi_{\mathrm{CF-RT}} \Big[ 1 + C (T-T_{0}) \Big]  $$

where *C* is the experimentally calculated temperature coefficient of resistivity and *φ*_*CF*−*RT*_ is the standard CF resistivity at room temperature.

COMSOL Multiphysics Software [147] is used for solving the coupled equations for this RRAM model due to its multiphysics capabities and ability to handle such simulations.

### Bocquet Bipolar Model

Bocquet bipolar model [148] describes the bipolar oxide-based resistive switching memories utilizing a physics-based modeling approach. Bocquet bipolar model describes the electroforming process of RRAM device, inaddition to utilizing some of the characteristics from Bocquet unipolar model [149] and modifies them significantly according to the bipolar switching characteristics. In this model, the radius of the CF is the internal state variable which effectively governs the switching rate.

To model the electroforming stage, Bocquet bipolar model utilizes electroforming rate (*τ*_Form_) which details the mechanism of conversion to switchable sub-oxide from pristine oxide. The electroforming stage is modeled as [148]:

20 $$ \tau_{\text{form}} = \tau_{\text{form}0} \times \exp \bigg({\frac{E_{a\text{Form}}-q \times \alpha_{s} \times V_{\text{cell}}}{k_{B}\times T}} \bigg)  $$

21 $$ {\frac{dr_{\text{CFmax}}}{dx}} = {\frac{r_{\text{work}}-r_{\text{CFmax}}}{\tau_{\text{form}}}}  $$

where *E*_*a*Form_ is the activation energy for electroforming, *τ*_form0_ is the nominal forming rate, *α*_*s*_ is the charge transfer coefficient, *V*_cell_ is the voltage applied between the top and bottom electrodes, *r*_CF_ is the radius of CF which varies from 0 to *r*_CFmax_, *q* is the elementary charge of electron, *T* is the temperature of the device and *k*_B_ is the Boltzmann constant.

The electrochemical redox reaction derived from Butler-Volmer equation [150] is used to describe the SET/RESET operation as [148]:

22 $$ \tau_{\text{Red}} = \tau_{\text{Redox}} \times \exp \bigg({\frac{E_{a}-q \times \alpha_{s} \times V_{\text{cell}}}{k_{B} \times T}} \bigg)  $$

23 $$ \tau_{Ox} = \tau_{\text{Redox}} \times \exp \bigg({\frac{E_{a}+q \times (1 - \alpha_{s}) \times V_{\text{cell}}}{k_{B} \times T}} \bigg)  $$

Here, *τ*_Red_ and *τ*_Ox_ are the reduction and oxidation rates, respectively. *τ*_Redox_ is the effective reaction rate considering both reduction and oxidation reactions.

The switching rate is obtained by coupling the above two equations as [148]:

24 $$ {\frac{dr_{CF}}{dt}} = {\frac{r_{\text{CFmax}}-r_{\text{CF}}}{\tau_{\text{red}}}} - {\frac{r_{\text{CF}}}{\tau_{\text{Ox}}}}  $$

Bocquet bipolar model is a quite comprehensive model as it includes the temperature effects as well. The local filament temperature is coupled using heat equation and is given in Eq.(25), the temperature considering a cylindrical-shaped filament is given in Eq. (26). The maximum temperature reached into CF at *x* = 0, the middle of the filament is given in Eq. (27) and the equivalent electrical conductivity in the work area (*σ*_*eq*_) is given in Eq. (28).

25 $$ \sigma_{x} \times F(x)^{2} = - k_{th}.{\frac{d^{2}T(x)}{dx^{2}}}  $$

26 $$ T(x) = T_{\text{amb}}+{\frac{V^{2}_{\text{cell}}}{2. L^{2}_{x}.k_{th}}} \bigg({\frac{L^{2}_{x}}{4}- x^{2}} \bigg) \sigma_{eq}  $$

27 $$ T = T_{\text{amb}}+{\frac{V^{2}_{\text{cell}}}{8. k_{th}}} \sigma_{eq}  $$

28 $$ \sigma_{eq} = \sigma_{CF}.{\frac{r^{2}_{\text{CF}}}{r^{2}_{\text{work}}}} - \sigma_{Ox}. {\frac{r^{2}_{\text{CFmax}}-r^{2}_{\text{CF}}}{r^{2}_{\text{work}}}}  $$

where (*σ*_*x*_) is the local electrical conductivity, *F*(*x*) is the local electric field, *σ*_*CF*_ is the electrical conductivity of the conductive filament, *k*_th_ is the thermal conductivity and *T*_amb_ is the ambient temperature.

It must be mentioned here that temperature increases with increase in radius of the CF, resulting in self-accelerated reaction due to a positive feedback loop. The self-limited reaction also referred to as SOFT reset [151], on the other hand, occurs due to the decrease in temperature and radius of the CF during RESET operation.

The total current flowing in OxRRAM is the sum of currents flowing in the conductive area (*I*_CF_), the conduction through switchable sub-oxide (*I*_sub−oxide_) and conduction through unswitched pristine oxide (*I*_pristine_). The total current is as [148]:

29 $$ I_{\text{cell}} = I_{\mathrm{sub-oxide}} + I_{\text{CF}} + I_{\text{Pristine}}  $$

30 $$ I_{\text{CF}} = F.\pi. \sigma_{CF}.r^{2}_{CF}  $$

31 $$ I_{\mathrm{sub-oxide}} = F.\pi. \sigma_{Ox}. \big(r^{2}_{\text{CFmax}}- r^{2}_{CF}\big)  $$

32 $$ I_{\text{Pristine}} = S_{cell}.A.F^{2}. \exp {\frac{-B}{F}}  $$

33 $$ A = {\frac{m_{e}.q^{3} }{8\pi.h.m^{ox}_{e}.\phi_{b} }}  $$

The parameter *B*_*e*_ is the metal-oxide barrier height (*ϕ*_*b*_)-dependent and is given as [148]:

$$ if \phi_{b}\geq qL_{x}F: B_{e} = {\frac{8 \pi \sqrt{2m^{ox}_{e} }}{3\times h\times q}} \Big[ \phi^{{\frac{3}{2}}}_{b}- (\phi_{b}-qL_{x}E)^{{\frac{3}{2}}} \Big] $$

34 $$ \text{otherwise}, B_{e} = {\frac{8 \pi \sqrt{2m^{ox}_{e} }}{3\times h\times q}} \times \phi^{{\frac{3}{2}}}_{b}  $$

where m _*e*_ and $m^{ox}_{e}$ are the effective electron masses into the cathode and oxide respectively, *F* = $\frac {V_{\text {cell}} }{L_{x}}$ is the electric field across the active layer, *h* is the Planck constant and *S*_cell_ is the section of the RRAM cell.

Discrete solutions are required to implement the model in an electrical simulator. This model accounts well in that aspect, making it suitable for simulation involving electrical circuits. This model implements equations in Eldo circuit simulator [152]. The discrete solutions are given as [148]:

35 $$ r_{\text{CFmax}_{i+1}} = \big(r_{\text{CFmax}_{i}}- r_{\text{work}} \big) \times e^{ {\frac{-\Delta t}{{\tau}_{\text{form}}}} } + r_{\text{work}}  $$

36 $$ r_{CF_{i+1}} = \bigg(r_{CF_{i}}- r_{\text{CFmax}_{i}} \times {\frac{\tau_{eq}}{\tau_{\text{Red}}}} \bigg) \times e^{ {\frac{-\Delta t}{{\tau}_{eq}}} } + r_{\text{CFmax}_{i}} \times {\frac{\tau_{eq}}{\tau_{\text{Red}}}}  $$

37 $$ \text{where} { \tau_{eq}} = \frac{\tau_{\text{Red}}\times \tau_{\text{Ox}} }{\tau_{\text{Red}}+\tau_{\text{Ox}}}  $$

This model has been verified against electrical characterization from an HfO_2_-based system [153]. An important feature of this model is that it can account effectively for device to device variability [154, 155]. One of the major limitations of this model is the lack of current or voltage threshold.

This section presents in detail various characteristics and features of the RRAM models. A comparative analysis of the RRAM models discussed in this work is presented in Table 5.

**Table 5:** Comparison of RRAM models

	ASU/Stanford [130]–[132]	Physical electro-thermal model [135]	Huang’s physical model [142]	Filament dissolution model [143]–[145]	Bocquet bipolar model [148]
Device type	Bipolar	Bipolar	Bipolar	Unipolar	Bipolar
State variable	Filament gap (g)	Ion concentration	Filament gap (g)	Ion concentration	Radius of CF
			and width of CF		
Control mechanism	Voltage	Voltage	Voltage	Voltage	Voltage
Simulation compatible	SPICE/Verilog	COMSOL	SPICE	COMSOL	SPICE

## Applications of RRAM

RRAM is seen as one of the standout candidates among the emerging memory technologies that has the potential for reforming the memory hierarchy primarily due to its high speed, the capability of non-volatile data storage, enhanced storage density and logic computing function. The various novel applications of RRAM are discussed in this section.

***>Non-volatile Logic***

The instruction codes and the data are transferred by making use of buses between various units in a computer system having von Neumann architecture because of the separate computing and memory unit. This data transferring process results increased energy consumption and time delay, which is commonly referred to as ‘von Neumann bottleneck’. For reducing the impact of von Neumann bottleneck [156], the computing process which utilizes RRAM crossbar array is suggested which alters the memory and computing operations in the same core. In addition, to obtain high integration density and low cost [157], two-terminal compact device structure of RRAM and its 4*F*^2^ array architecture are highly beneficial. For example, to obtain simple Boolean logic functions such as ‘logic NOT’, ‘logic AND’, and ‘logic OR’, we require multiple transistors and each single transistor takes 8−10*F*^2^ area. These logic functions can be realized by making use of two or three RRAM cells, resulting in total approximate area of around 10*F*^2^ only [158].

Till date, several methods have been suggested for realizing Boolean logic functions [159, 160]. Boolean computing is significantly more established compared to existing non-Boolean computing paradigms such as neuromorphic computing and quantum computing. Therefore, energy and cost-efficiency of CPU or MCU can be enhanced without the need to develop new algorithms or software, although there is still a lack of technical solution on how to implement complex computing tasks in a crossbar array. Thus, most of research to date focusses on only basic logic level demonstration as it becomes quite complex to implement a whole computing unit using RRAM array.

***Neuromorphic Computing***

To overcome ‘von Neumann bottleneck’, one of the effective ways is brain-inspired neuromorphic computing which has shown promising potential in a wide range of complex and cognitive tasks like visual/audio recognition, self-driving, and real-time big-data analytics. Compared to CMOS-based neuromorphic network, neuromorphic computing based on RRAM-array offers advantages in terms of on-chip weight storage, online training, and scaling up to much larger array size [161–163]. In addition, the processing speed of RRAM improves by three orders of magnitude, whereas the power consumption rate is reduced by four orders of magnitude [164].

For realizing hardware-implemented neuromorphic computing paradigms, two methods are suggested: one among the strategies mimics the structure and working mechanism of biological neural networks while the other method works on accelerating the existing artificial neural network (ANN) algorithms. In a neural network, a synapse is used to transfer spikes between different neurons in addition to storing information about the transferring weights. The information regarding weights can be acquired through certain learning rules such as spike-time-dependent plasticity (STDP) and spike-rate-dependent plasticity (SRDP) [165–167]. Although some of the works reported in the literature have tried to emulate such learning rules on RRAM devices, it is however quite complicated to extend such types of bioinspired learning rules to a complex task as the theoretical algorithm is still lacking.

A practically viable approach is to map an ANN to a RRAM-based neuromorphic network directly. Some advanced tasks such as pattern and speech recognition have been demonstrated based on this method [166–169]. Although very promising, RRAM-based synapse is still far from being applied as various issues such as material optimization, variation suppressing, control circuit design, architecture, and algorithms design for analog computing need to be addressed effectively.

***Security Application***

The security aspect has become more prominent with rapid developments in the field of information technology; thus, there is a need for hardware-based security-integrated circuits. In contrast to security circuits based on CMOS logic which exploits the random nature of the semiconductor manufacturing process, security circuits based on RRAM are more robust to attacks of various types due to its completely random switching mechanisms [170, 171]. It must be noted that for security applications, larger variation of RRAM device parameters such as random telegraph noise (RTN), resistance variations and probabilistic switching is desirable, which is quite different from memory applications that require a smaller degree of variation among numerous parameters.

A novel security feature commonly referred to as physical unclonable function (PUF) [172], based on RRAM is proposed for device authentication (strong PUF) and key generation (weak PUF) applications. Significantly larger number of input-output pairs [also called challenge-response pair (CRP) are required for strong PUF, while only a small amount of CRPs of extremely higher reliability are required for weak PUF [173]. Although, PUFs based on RRAM have demonstrated remarkable performance; however, still more practical demonstrations and further evaluations are required to work out the maturity of this new primitive within the field of hardware security.

***Non-volatile SRAM***

Volatile memory technologies like SRAM and DRAM may consume over half of the static power within the current mobile SoC chips. Thus, to attain fast parallel memory operations, reduced area and low-energy consumption, RRAM-based non-volatile SRAM (nvSRAM) was proposed [174] in which two RRAM cells are stacked on eight transistors, forming an 8T2R structure. Also, non-volatile ternary content-addressable memory (TCAM) having 4T2R cell structure [175] and non-volatile flip flops having reduced stress time and write power based on RRAM have been demonstrated recently [176].

## Challenges and Future Outlook

During the past several years, research in the field of emerging memory technologies has grown significantly and several prototype RRAM products have been developed demonstrating the potential for high-speed and low-power embedded memory applications. RRAM is one of the most promising memory technologies because of the advantages of simple structure, compatibility with the existing CMOS technology, good switching speed and ability to scale to the smallest dimensions. As a matter of fact, currently the Flash memory technology is facing difficulties to reduce to lower dimensions and as such RRAM is emerging as a potential replacement especially for fast operation and medium size storage density memory applications.

One of the most critical aspects that needs to be thoroughly investigated is that of the reliability of RRAM. A mechanism must be developed to ensure the detection of the operation failure of the device. Also designing circuits, e.g. a test element group (TEG) design for robust signal sensing, is one of the critical challenges for the emergence of RRAM devices. To achieve high-density memory operation in RRAM, the 1D1R operation is essential. This can be realized by operating the RRAM device in the unipolar mode. However, in the unipolar operation, higher current is needed for the reset process as compared to that of the bipolar operation. This is due to the fact that thermal effect plays a significant role in the unipolar reset process. Thus, to realize a high-density 1D1R RRAM array, the thermal effects both inside and outside a memory element needs to be considered. Also note that till date, in a single RRAM device, no technology has simultaneously reported fast switching, low power, and stable operation. Although, the endurance of RRAM has been reported as high as 10^12^ [59], it is still not enough to be able to replace DRAM. The RRAM possesses the switching speed fast enough for DRAM replacement and the materials used in the fabrication for RRAM are very similar to that of DRAM, it becomes a critical challenge to improve the endurance characteristics of RRAM. To improve the endurance characteristics, it is necessary to control the oxygen movement between the electrode and the oxide layer at the interface. It is suggested to insert the second metal layer at the interface which can be easily oxidized and acts as an oxygen reservoir to prevent oxygen from penetrating into the electrode during the resistance switching. The most critical challenge hindering RRAM development till date is the proper understanding of the device switching mechanism which is since long being debated by researchers across the globe. The inconsistent switching mechanism of various reported RRAM devices is believed to be because of variation in the fabrication process, and thus, more rigorous analysis is needed in the future for obtaining a better understanding of the switching mechanism of RRAM devices. The aforesaid issues need to be handled effectively before implementing RRAM in future memory applications. Although, RRAM is highly attractive for use in neuromorphic computations, the biggest challenge to industrialize RRAM lies in its ability to tackle the variability issues, not only at nominal operating conditions but also at high temperatures before they can be used in a wide variety of applications.

## Conclusion

This review article provides a brief introduction into the advancement of the memory architecture, the current trends and the limitations while providing a valuable insight into the field of emerging memory technologies. A detailed discussion, highlighting the importance of RRAM, its structure, working mechanism, and classification, has been presented. The key performance parameters and their effect on the RRAM operation has also been detailed within the current manuscript. An elaborate study on the MLC capability of RRAM, along with the methodology have been presented. The manuscript also discusses the important features of the widely accepted RRAM models. The implementation of RRAM for various important applications such as non-volatile logic, neuromorphic computing, security, and non-volatile SRAM have been highlighted. Although, significant success has been achieved in RRAM technology; however, more work is needed as RRAM still suffers from various challenges in terms in terms of high operation current, lower resistance ratios, and reliability issues. More efforts in research should aim to develop methods to achieve faster programming/erasing, lower power consumption, enhancing the storage density by implementing multilevel storage capability and improvement in the fabrication process for enhanced uniformity. In addition, renewed focus should be towards use of RRAM in embedded memory and non-volatile logic applications as breakthroughs in these fields are much more exciting and significant. With continued work and improvements, it is imperative that RRAM devices will be a standout technology for future non-volatile memory applications.

## Data Availability

Not applicable.

## Abbreviations

RRAM: Resistive random access memory
MLC: Multilevel cell
RTN: Random telegraph noise
DRAM: Dynamic random access memory
SRAM: Static random access memory
PCM: Phase change memory
STT-MRAM: Spin-transfer torque resistive random access memory
LRS: Low resistance state
HRS: High resistance state
MTJ: Magnetic tunneling junction
MIM: Metal-insulator-metal
MoM: Metal-oxide-metal
PLD: Pulse laser deposition
ALD: Atomic layer deposition
*V*_*set*_: Set voltage
*V*_*reset*_: Reset voltage
*V*_*f*_: Forming voltage
*I*_CC_: Compliance current
CBRRAM: Conductive bridge resistive random access memory
OxRRAM: Oxygen vacancies resistive random access memory
ECM: Electrochemical metallization memory
VCM: Valence change memory
CF: Conductive filament
BE: Bottom electrode
TEM: Transmission electron microscopy
I-V: Current-voltage
1T-1R: 1-Transistor 1-RRAM
ANN: Artificial neural network
STDP: Spike-time-dependent plasticity
SRDP: Spike-rate-dependent plasticity
PUF: Physical unclonable function
CRP: Challenge-response-pair
nvSRAM: Non-volatile SRAM
TCAM: Ternary content addressable memory
TEG: Test element group
